# SLC2A9 rs16890979 reduces uric acid absorption by kidney organoids

**DOI:** 10.3389/fcell.2023.1268226

**Published:** 2024-01-10

**Authors:** Shouhai Wu, Chuang Li, Yizhen Li, Junyi Liu, Cuiping Rong, Hongfei Pei, Xiong Li, Xiang Zeng, Wei Mao

**Affiliations:** ^1^ State Key Laboratory of Dampness Syndrome of Chinese Medicine, The Second Affiliated Hospital of Guangzhou University of Chinese Medicine, Guangzhou, China; ^2^ Department of Nephrology, Guangdong Provincial Hospital of Chinese Medicine, Guangzhou, China; ^3^ Guangdong Provincial Key Laboratory of Chinese Medicine for Prevention and Treatment of Refractory Chronic Diseases, Guangzhou, China; ^4^ The Second Clinical Medical College, Guangzhou University of Chinese Medicine, Guangzhou, China; ^5^ Lab of Stem Cell Biology and Innovative Research of Chinese Medicine, Guangdong Provincial Hospital of Chinese Medicine/Guangdong Academy of Chinese Medicine, Guangzhou, China; ^6^ National Institute for Stem Cell Clinical Research, Guangdong Provincial Hospital of Chinese Medicine/The Second Affiliated Hospital of Guangzhou University of Chinese Medicine, Guangzhou, China

**Keywords:** uric acid, SLC2A9/GLUT9, kidney organoids, CRISPR/Cas9, hyperuricemia, hypouricemia

## Abstract

**Introduction:** The excretion and absorption of uric acid (UA) by the kidneys helps regulate serum UA levels. GLUT9, encoded by *SLC2A9*, is mainly expressed in the renal tubules responsible for UA absorption. *SLC2A9* polymorphisms are associated with different serum UA levels. However, the lack of proper *in vitro* models has stalled research on the mechanisms of single nucleotide polymorphisms (SNPs) that affect UA metabolism in human urate transporters.

**Methods:** In this study, we constructed a gene-edited human embryonic stem cells-9 (ESC-H9) derived kidney organoid bearing rs16890979, an *SLC2A9* missense mutation with undetermined associations with hyperuricemia or hypouricemia. Kidney organoids derived from ESC-H9 with genetical overexpression (OE) and low expression (shRNA) of *SLC2A9* to serve as controls to study the function of *SLC2A9*. The function of rs16890979 on UA metabolism was evaluated after placing the organoids to urate-containing medium and following histopathological analysis.

**Results:** The kidney organoids with heterozygous or homozygous rs16890979 mutations showed normal *SLC2A9* expression levels and histological distribution, phenotypically similar to the wild-type controls. However, reduced absorption of UA by the kidney organoids with rs16890979 mutants was observed. This finding together with the observation that UA absorption is increased in organoids with *SLC2A9* overexpression and decreased in those with *SLC2A9* knockdown, suggest that GLUT9 is responsible for UA absorption, and the rs16890979 SNP may compromise this functionality. Moreover, epithelial-mesenchymal transition (EMT) was detected in organoids after UA treatment, especially in the kidney organoid carrying GLUT9^OE^, suggesting the cytobiological mechanism explaining the pathological features in hyperuricosuria-related renal injury.

**Discussion:** This study showing the transitional value of kidney organoid modeling the function of SNPs on UA metabolism. With a defined genetic background and a confirmed UA absorption function should be useful for studies on renal histological, cellular, and molecular mechanisms with this organoid model.

## Graphical Abstract

**Figure:**
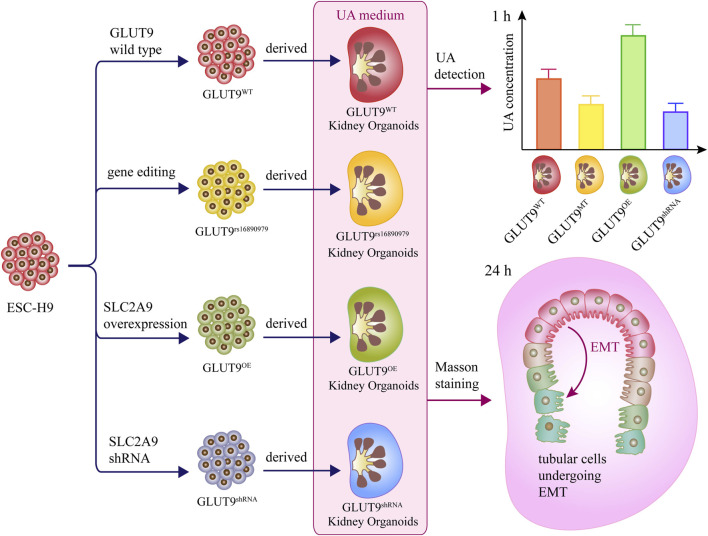
The excretion and absorption of uric acid (UA) by the kidneys helps regulate serum UA levels. GLUT9, encoded by *SLC2A9*, its polymorphisms are associated with serum UA levels. However, there is still a lack of ideal cellular and animal models to study the impact of single nucleotide polymorphisms in human urate transporters on UA metabolism. Here we constructed a gene-edited embryonic stem cell-derived kidney organoid bearing rs16890979, an *SLC2A9* missense mutation which affects serum UA levels. We found that the kidney organoids with rs16890979 mutants showed reduced absorption of UA. This finding together with the observation that UA absorption is increased in organoids with *SLC2A9* overexpression and decreased in those with *SLC2A9* knockdown, suggesting that *SLC2A9* is responsible for UA absorption, and the rs16890979 may compromise this functionality. Moreover, we detected epithelial-mesenchymal transition in the organoids when placed them into urate-containing medium for 24 h; this may represent the cytobiological mechanism explaining the pathological features in hyperuricosuria-related renal injury. Therefore, our results prove the value of the kidney organoid model in the study of the function of SNPs on UA metabolism.

## 1 Introduction

Uric acid (UA) metabolism abnormalities, including UA under-excretion or under-absorption by the kidneys are the main cause of hyperuricemia (HUA) and hypouricemia, respectively; and, they lead to a variety of renal-related diseases such as gout or exercise-induced acute kidney injury (Chiba et al., 2014; Takahashi et al., 2021). One of the pathological features of HUA induced renal injury is the occurrence of EMT in the kidneys, which later contributes to renal fibrosis (Balakumar et al., 2020). Studies have shown that HUA might trigger EMT by activating signaling pathways such as NRLP3 and NFκB (Liu et al., 2015; Dalbeth et al., 2021; Wu et al., 2023). UA within the primary urine is reabsorbed into the proximal convoluted tubule cells through reabsorption transporters that include URAT1 (*SLC22A12*) and GLUT9 (*SLC2A9*) (Anzai et al., 2008; Vitart et al., 2008). GLUT9 is mainly expressed in the kidneys, liver, and chondrocytes, and its initially discovered function was as a glucose transporter (Phay et al., 2000; Mobasheri et al., 2013). GLUT9 has two splice variants, GLUT9a and GLUT9b (GLUT9DeltaN). GLUT9a localizes predominantly on the basolateral surface and GLUT9b on apical surface of the epithelium of renal tubes (Augustin et al., 2004; Doblado and Moley, 2009). These two variants synergistically recycle UA from the primary urine into the blood system. Genetic polymorphisms affecting renal UA metabolism, mainly single nucleotide polymorphisms (SNPs) in urate transporters, have been described (Kottgen et al., 2013; Tin et al., 2019). For example, a genome-wide association study (GWAS) and candidate gene analyses suggested that serum UA levels are associated with SNPs within the *SLC2A9* gene (Matsuo et al., 2008; Dinour et al., 2010; Dinour et al., 2011). The variation within disease associations also extends to the link between GLUT9 SNPs and gout (Hollis-Moffatt et al., 2009). However, GWAS are retrospective in nature and may detect risk gene variants, but not necessarily causal genes responsible for underlying genetic variations in serum UA levels (Tin et al., 2019). Indeed, many factors other than specific SNPs including ethnicity, sex, and comorbidities (Döring et al., 2008) may affect *SLC2A9* functions, leading to variations in serum UA levels. There are reports on the effects of uric acid transporters (such as URAT1) on UA absorption (Li et al., 2019). However, the appropriate cell or animal models for study the urate transporter SNP mechanisms affecting UA metabolism in humans are still lacking. In addition, this gap in the relevant human genetic background knowledge has slowed drug development advancements (Keenan, 2020; Halperin Kuhns and Woodward, 2021).

The rapid progress in kidney organoid development has resulted in a model able to simulate major kidney functions. For example, the expression of renal-associated UA transporters (including GLUT9) among other compositional and functional proteins was validated in a human pluripotent stem cell-derived kidney organoid model (Wu et al., 2018), offering the possibility of studying the effects of *SLC2A9* SNPs on UA absorption *in vitro*. However, kidney organoids with manipulated SNPs for UA metabolism study are not yet available.

For this study, we established kidney organoids from human embryonic stem cells-9 (ESC-H9) with heterozygous (H9-SLC2A9^monoallelicMT^) or homozygous (H9-SLC2A9^biallelicMT^) rs16890979 SNPs using the CRISPR/Cas9 technique. rs16890979 is a well-documented missense single-base G to A mutation in exon 8 of *SLC2A9*, leading to the replacement of the valine for an isoleucine in position 282 for GLUT9a and 253 for GLUT9b, respectively. As mentioned above, a GWAS indicated that rs16890979 is associated with UA metabolism (McArdle et al., 2008; Parsa et al., 2012). However, reports have shown that rs16890979 correlates with either hyperuricemia or hypouricemia depending on ethnic and geographic variations (Dehghan et al., 2008; McArdle et al., 2008; Meng et al., 2015; Cho et al., 2020). Thus, the exact significance of rs16890979 needs an in-depth cytobiological clarification. We applied gene editing and developmental biology techniques to assess the effect of rs16890979 on UA absorption in human kidney organoids with a defined genetic background. Our proof-of-concept experimental approach may become a paradigm to study the biological function of SNPs on UA metabolism.

## 2 Methods

### 2.1 Cell culture, self-renewal, and cell growth assays

Human H9 embryonic stem cell line cell (hESC, H9) (the 11th passage) was provided by Professor Yang Xu from the Center for Regenerative and Translational Medicine of our Affiliation (Liu et al., 2019). In theory, ESC has a pluripotent differentiation ability. Here, we referred to the method in reference (Morizane et al., 2015) and selected H9 cells for research. We cultured H9 on mouse embryonic fibroblast feeder layers in knockout Dulbecco’s modified Eagle’s medium (DMEM; Life Technologies, Carlsbad, CA, United States) supplemented with 20% knockout serum replacement (Invitrogen, Carlsbad, CA, United States), 1% PenStrep (Invitrogen, Carlsbad, CA), 1% Glutamax (Invitrogen, Carlsbad, CA), 1% nonessential amino acids (Invitrogen, Carlsbad, CA, United States), 10 ng/mL bFGF, and 55 μM *ß*-mercaptoethanol. For the feeder-free culture of hESCs, we maintained the hESCs on Matrigel-coated plates in mTeSR1 medium (Stem Cell Technologies, Vancouver, BC, Canada). Typical morphology for the cultured hESC through like the dense cell clones with smooth edges was confirmed, and pluripotency markers like TRA-1-81 and OCT4 were detected by immunofluorescence staining. We cultured HEK293T cells in DMEM (Gibco, Carlsbad, CA, United States) medium supplemented with 10% fetal bovine serum (FBS; Hyclone, Logan, UT, United States), and 1% PenStrep (Invitrogen, Carlsbad, CA, United States). All cells were cultured in a 37°C incubator with 5% CO_2_.

### 2.2 Plasmids construction

We achieved precise gene targeting homologous recombination using a CRISPR/Cas9 nickase in hESC-H9 cells with a long DNA donor template. Several guide RNAs (gRNA) of 200–400 bp containing the mutation site were ligated into Cas9-P2A (addgene, #99248) plasmids. We verified the cutting efficiency of gRNAs using a *T7E1* enzyme (NEB, #E3321) and we chose the gRNA (sequence GCC​ACA​AAC​TCT​ATG​CTG​TTA​GG) with the best cutting efficiency to design the repair template. The position of the gRNA was in the intron downstream of the exon 8 of GLUT9 (encoding both GLUT9a and GLUT9b; the plasmid map was shown in Figure 2C), with a distance of 110 bp from the exon 8 to avoid alteration of the sequence in the exon. Therefore, gene editing did not affect the transcriptional regulation of the exon 8. To construct the wild type repair template, we amplified the upstream 481-bp fragment containing the mutation point and the downstream 202-bp fragment of the repair template by PCR using DNA from H9 as a template. Table 1 lists the PCR primers used. We used *NotI* (NEB, R3189L) and *EcoRI* (NEB, R3101L) restriction enzymes to linearize the double-cut pBluescriptKS plasmid (addgene, #104475); and, after separating the resulting DNA bands after electrophoresis in 1% EB gel, we extracted a ∼2900-bp fragment. Next, we also digested the upstream 481-bp fragment using *NotI* and *EcoRI* and ligated it to pBluescriptKS using T4 ligase to obtain upstream-pBluescriptKS, and we digested the upstream-pBluescriptKS and the downstream 202-bp fragment using *XhoI* (NEB, R0146L) and *KpnI* (NEB, R3142L) and ligated the fragments with *T4* ligase (Promega, M1794). After that, we amplified the same fragments using primers with mutated bases (Forward 5′- CGT​TCT​TGG​GTA​AAG​CAG​AC a TTT​CCC​AAG​AGG​TAG​AGG​AG -3′ and Reverse 5′- CTC​CTC​TAC​CTC​TTG​GGA​AA t GTC​TGC​TTT​ACC​CAA​GAA​CG -3′) by PCR using high fidelity enzyme, followed by *Dpnl* (NEB, R0176S) digestion to remove the methylated plasmid, and proceeded to the transformations into DH5α cells. We selected reaction clones after sequencing. For the construction of the SLC2A9-overexpression plasmid, we performed gene cloning and PCR amplification of SLC2A9-mRNA from H9-Cell genomic DNA using the primers listed in Table 1. The SLC2A9-mRNA downstream fragment was inserted after the CMV promoter into a pLenti vector (pLenti-CMV-GFP-Puro, addgene-17448) using the *XbaI* (NEB, R0145L) and *SalI* (NEB, R3138L) restriction enzyme sites. For the construction of the SLC2A9-shRNA plasmid for SLC2A9-downregulation, we selected the target sequence GGA​CGT​TAA​TTG​TGA​AGA​TGA and digested the primers (Table 1) and the pLKO.1 vector using the restriction enzymes *AgeI* (NEB, R3552L) and *EcoRI* and ligated them using *T4* ligase. All the plasmids were transformed it into DH5α cells, cultured on Luria-Bertani medium with ampicillin and then sequenced. We selected a clone with the correct sequence to amplify and extract the fragments for the following experiments. Table 1 lists the primer sequences used.

**TABLE 1:** Primer list for plasmids constructed.

Primers for plasmid construction (F: Forward (5′to 3′), R: Reverse (5′to 3′))	
SLC2A9-uparm-F	ata​gcg​gcc​gcT​AAA​CAA​GGG​CTT​TGG​ACT (NotI)
SLC2A9-downarm-R	ccg​gaa​ttc​AAC​AGC​ATA​GAG​TTT​GTG​GC (EcoRI)
SLC2A9-downarm-F	ccg​ctc​gag​AGG​TAA​CCA​TGT​AAC​TTG (XhoI)
SLC2A9-downarm-R	cgg​ggt​acc​CTT​CTG​ACT​TCC​TAA​TAT​C (KpnI)
SLC2A9-SNP-Seq-F	TGCTCTGCCTTGCGTTGC
SLC2A9-SNP-Seq-R	TGAACCTGGGCGTCTGG
SLC2A9-OE-F	ccc​ctc​tag​aAT​GAA​GCT​CAG​TAA​AAA​GGA​CCG (XbaI)
SLC2A9-OE-R	ccc​cgt​cga​cTT​AAG​GCC​TTC​CAT​TTA​TCT​TA (SalI)
SLC2A9-shRNA-F	ccg​gGG​ACG​TTA​ATT​GTG​AAG​ATG​Act​cga​gTC​ATC​TTC​ACA​ATT​AAC​GTC​Ctt​ttt​ttg
SLC2A9-shRNA-R	aat​tca​aaa​aaa​GGA​CGT​TAA​TTG​TGA​AGA​TGA​ctc​gag​TCA​TCT​TCA​CAA​TTA​ACG​TCC

### 2.3 Lentivirus production and cell transfection

We generated lentiviral *SLC2A9* overexpression (OE) and shRNA vectors by inserting the *SLC2A9* coding sequence or small interfering RNA sequence downstream of the pLenti CMV-GFP-Puro or pLKO.1 promoter. The scramble control vectors, OE or shRNA vector were separately cotransfected with psPAX2 and pMD2. G plasmids (Addgene, Watertown, MA, United States) into HEK293T cells using a lipofectamine 3,000 reagent (Thermo Fisher, L3000008). Forty-8 hours after the transfections, we collected the supernatants and used a Lenti-X concentrator (Clontech, Mountain View, CA, United States) to isolate the viruses. We then transfected H9 cells with either scramble or *SLC2A9* shRNA lentivirus particles and selected colonies growing onto 0.5 μg/mL puromycin plates.

To construct hESCs with *SLC2A9* point mutations, we transfected gRNA-Cas9-P2A and template at a molar ratio of 1:3 into H9 cells using a LONZA-4D cell nuclear electrotransfection apparatus with the P3 Primary Cell 4D-Nucleofector X CB-150 kit. We picked positive single clones of H9 cells after puromycin selection, and we verified the clone sequences by PCR. The Cre/Lox (pDZ416 loxP-Kan-loxP, addgene #45163) plasmid was then transfected to remove the fragment of Loxp-puro-loxp, and we verified the correct clone by Sanger sequencing. The gene editing did not alter the sequence of the eighth exon of GLUT9. After editing, both variants, GLUT9a and GLUT9b, presented an isoleucinein stead of the valine. H9 cells with the verified mutation sites of homozygous and heterozygous H9-GLUT9^rs16890979^ were termed H9-GLUT9^monoallelicMT^ and H9-GLUT9^biallelicMT^, respectively.

### 2.4 Organoid formation

Prior to differentiation, we cultured H9 hESCs in 6-cm diameter plates with medium to reach approximately 80% confluence. On day 0, cells were dissociated with 1 mg/mL of Dispase II (Gibco, 17105041), centrifuged, and resuspended in BPEL (BSA poly (vinyl alcohol) essential lipids) medium supplemented with 3.3 mM Y27632 (Sigma-Aldrich, 129830-38-2), 8 μM CHIR99021 (Sigma-Aldrich, SML1046), and 1 mM *ß*-mercaptoethanol (Gibco, 21985023). The cell suspensions were subsequently cultured in 6-well ultra-low concentration attachment plates (Corning, NY, United States). On day 2, we replaced half of the medium with BPEL with CHIR99021 (8 μM). On day 3, embryoid bodies (EBs) were formed. After that, the EBs were transferred into phase II medium (IMDM and Ham’s F-12 nutrient mixture) with half-volume medium replacements every 2 days for a total 14 days of differentiation, as described (Przepiorski et al., 2018).

### 2.5 UA medium configuration and UA detection

The components of the UA medium used were based on the STEMdiff™ Kidney Organoid Kit (STEMCELL# 05160) and 1,000 μM urate (Shanghai Yuanye Bio-Technology, China). We determined the urate concentrations to use by searching the literature for *in vitro* studies (Yan et al., 2019). We selected kidney organoids of similar size to minimize within/between-group errors. After 1, 2, 3, or 24 h of UA treatment, the organoids were gently rinsed with ice-cold PBS rapidly. To enhance the sensitively of the test, we resuspended five organoids grown under the same condition in 100 µL of ice-cold PBS and obtained a homogeneous sample by ultrasonication for 30s. The lysate was centrifuged at 12,000×*g* and 4°C for 5 min and the supernatant was collected for UA detection. Five μL of supernatant were added to 300 μL of buffer containing 50 mM Tris (pH 7.5, Sigma-Aldrich) and 1 mM sodium phosphate (Wako Pure Chemical Industries). We determined UA levels in the kidney organoids using a uricase method (Uric acid C-test Wako, Wako Pure Chemical Industries) (Adachi et al., 2017).

### 2.6 Homologous modeling and molecular docking of homo SLC2A9 (GLUT9)

For the homologous modeling of human *SLC2A9* (GLUT9) protein. The structures of wild type and mutated human *SLC2A9* (GLUT9) proteins were constructed by homologous modeling on the SWISS-MODEL (Waterhouse et al., 2018). The template for GLUT9a (NP_064425.2) and its mutant is PDB: 4YBQ, with sequence identity and coverage of 45.28% and 86.11%, respectively. The obtained structure was tested using Ramachandran plot. In the structure of GLUT9a^WT^ and GLUT9a^rs16890979^, the proportions located of ψ-φ in the Allowed, Marginal, and Disallowed regions are 98.2%, 1.5%, and 0.2%, respectively, indicating that the structures obtained through homologous modeling are basically reasonable. The template for GLUT9b (NP_001001290.1) and its mutant is PDB: 4YBQ, with sequence identity and coverage of 44.82% and 93.84%, respectively. The obtained structure was tested using Ramachandran plot. In the structure of GLUT9b^WT^ and GLUT9b^rs16890979^, the proportions located of ψ-φ in the Allowed, Marginal, and Disallowed regions are 99.8%, 0.2%, and 0.0%, respectively.

Then we performed molecular docking detection between UA (PubChem CID: 1175) molecules and GLUT9s. The docking between UA and GLUT9 was performed using AutoDock 4.2.6 (Morris et al., 2009). The binding site of GLUT9a^WT^ and GLUT9a^rs16890979^ structure was defined as a cube centered on VAL282 or ILE282 with a side length of 30 Å; while the binding site of GLUT9b^WT^ and GLUT9b^rs16890979^ structure was defined as a cube centered on VAL253 or ILE253 with a side length of 30 Å. The conformational optimization algorithm adopts Lamarck’s genetic algorithm and its default parameters.

### 2.7 Immunofluorescence

The H9 cells or kidney organoids were fixed with paraformaldehyde (4%) in PBS for 30 min at room temperature (RT). The organoids were dehydrated with sucrose (30% w/v in PBS) overnight at 4°C, embedded in optimal cutting temperature compound (Tissue-Tek, Torrance, CA, United States), and sliced into 10-μm thick sections using a cryostate. For immunofluorescence staining, we washed cells or sections three times with PBS (5 min each time), then incubated them in blocking buffer (5% BSA and 0.2% Triton X-100 in PBS) for 2 h at RT. After blocking, the sections were incubated with primary antibodies at 4°C overnight. The primary antibodies and their dilutions were the following: TRA-1-81 (MAB4381, Sigma-aldrich, 1:200), OCT4 (611202, BD Pharmingen, 1:200), NPHS1 (AF4269-SP, R&D systems, 1:200), CD31 (555444, BD, 1:200), LRP2 (ab76969, Abcam, 1:200), UMOD (SAB1400296-50UG, Sigma, 1:200), GATA3 (MAB6330, R&D, 1:200), SLC2A9 (Invitrogen, PA5-22960), LTL (Vector Labs, FL-1321), and MEIS1/2/3 (39796, Active Motif, 1:200). After washing the sections in PBS for 3 times, we incubated them with Alexa-Fluor conjugated secondary antibodies (Invitrogen) for 1 h at RT. The nuclei were counterstained with DAPI for 10 min. Slides were sealed with 80% glycerol, and we obtained images using a Zeiss LSM 710 confocal microscope equipped with ZEN software (ZEN 2011; Oberkochen, Germany).

### 2.8 Quantitative RT-PCR (qRT-PCR)

We extracted total RNA from cells/organoids using the RNeasy Plus Kit (Qiagen) according to the manufacturer’s instructions. For qRT-PCR analysis, we generated cDNA using a High Capacity cDNA Reverse Transcription Kit (Thermo Fisher Scientific) and performed qRT-PCR using the iTaq universal SYBR Green (Bio-Rad) on a CFX-96 Real-Time PCR Detection System (Bio-Rad). All data were normalized according to the *GAPDH* expression levels. Table 2 lists all the primers used.

**TABLE 2:** Related to mRNA expression test, Primer list for qRT-PCR.

Gene	Forward (5′to 3′)	Reverse (5′to 3′)
*GAPDH*	CGG​AGT​CAA​CGG​ATT​TGG​TC	GAC​AAG​CTT​CCC​GTT​CTC​AG
*SLC12A1*	AGT​GCC​CAG​TAA​TAC​CAA​TCG​C	GCC​TAA​AGC​TGA​TTC​TGA​GTC​TT
*NPHS1*	AGT​GTG​GCT​AAG​GGA​TTA​CCC	TCA​CCG​TGA​ATG​TTC​TGT​TCC
*GATA3*	GCC​CCT​CAT​TAA​GCC​CAA​G	TTG​TGG​TGG​TCT​GAC​AGT​TCG
*LRP2*	AAA​TTG​AGC​ACA​GCA​CCT​TTG​A	TCT​GCT​TTC​CTG​ACT​CGA​ATA​ATG
*CALB1*	TCC​AGG​GAA​TCA​AAA​TGT​GTG​G	GCA​CAG​ATC​CTT​CAG​TAA​AGC​A

### 2.9 Western blotting

We harvested kidney organoids in Pierce RIPA (Thermo-Fisher Scientific) buffer with 1% protease and 1% phosphatase inhibitors (Thermo-Fisher Scientific). The samples were sonicated for 1 min and then centrifuged at full speed for 10 min at 4°C. Cell sediments were discarded. We calculated protein concentrations using Pierce BCA Protein assays (Thermo-Fisher Scientific) and a BioTech plate reader per manufacturer instructions. We added 4 × loading buffer (Takara-9173) to each sample and boiled them at 100°C for 5 min. Proteins were transferred to PVDF membranes (Bio-Rad) and further incubated with the appropriate antibodies (SLC2A9, Invitrogen, PA5-22960; Vimentin, Abcam, ab92547; E-Cadherin, Abcam, ab231303; GAPDH, CST, #5174). We used Immobilon Western Chemiluminescent HRP Substrate (Millipore) for protein detection.

### 2.10 Electron microscopy

We fixed the organoids in 2.5% glutaraldehyde solution (Ala Aesar, A17876), then rinsed them with 0.1 M phosphate buffer (Sigma, P3813) before fixing them with 1% OsO_4_ (TED PELLA, 18456), rinsing them with 0.1 M phosphate buffer at 4°C, dehydrating them with ethanol, and embedding them in epoxy resin. We cut ultra-thin sections (50–70 nm) using ultrathin slicer (Leica UC-7) and stained them with uranyl acetate and lead citrate. The sections were then imaged using a transmission electron microscope (Japan Electron Optics Laboratory, JEM-1400) with acquiring image software (RADIUS ALL 2.2).

### 2.11 Statistical analysis

We performed all statistical analyses using GraphPad Prism (GraphPad Software, La Jolla, CA, United States). We used Student’s t-test for comparisons between two groups and applied an *F*-test to evaluate variance homogeneity. For comparisons between multiple groups, we used one-way analysis of variance (ANOVA) or two-way ANOVA. The figure legends include the sample size *n*). Independent studies were repeated at least 3 times. * indicates a *p* ≤ 0.05; ** indicates a *p* ≤ 0.01; *** indicates a *p* ≤ 0.001; ns indicates no statistical significance. Error bars represent mean ± SD.

## 3 Results

### 3.1 Kidney organoids derived from H9 human embryonic stem cells

**FIGURE 1:**
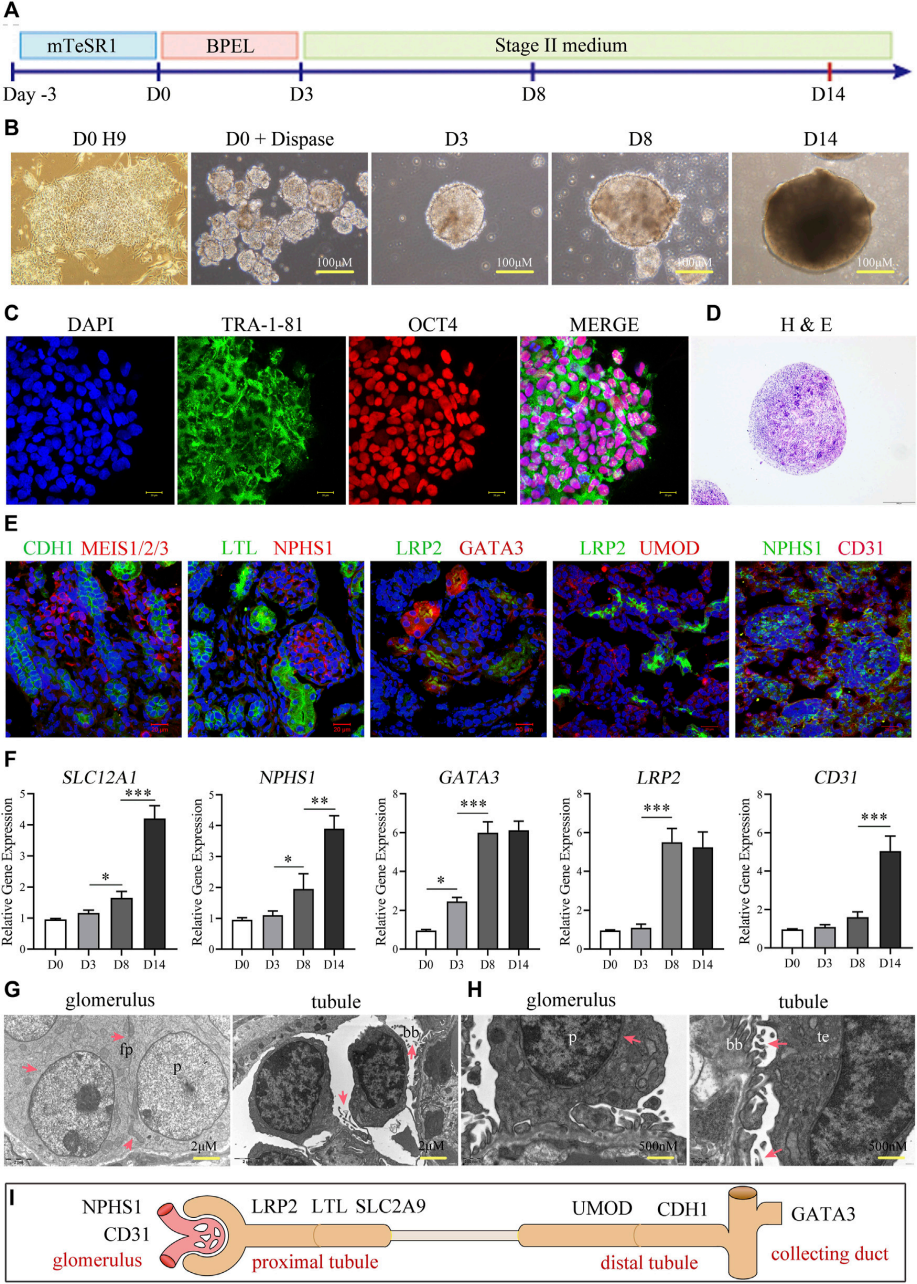
Construction and characterization of kidney organoids. **(A)** Schematic diagram showing the stepwise induction of kidney organoids from H9 ESCs. **(B)** Morphology of H9 cells before organoid induction (day 0), H9-derived embryoid bodies, and cell assemblies/organoids at days 8 and 14 after induction, respectively. **(C)** Representative colonies of passage-30 H9 cells with immunostaining for the pluripotency-associated markers TRA-1-81 and OCT4 (scale bar = 20 μm). **(D)** H&E staining showing tubular profiles and multiple morphologies of cell assemblies within organoids sectioned at day 14. **(E)** Immunostaining of renal cell markers CDH1, MEIS1/2/3, LTL, NPHS1, LRP2, GATA3, UMOD, and CD31 (Scale bar = 20 μm, ×40 oil objective). **(F)** Temporal expressions of *SLC12A1*, *NPSH1*, *GATA3*, *LRP2,* and *CD31* analyzed by qRT-PCR at days 0, 3, 8, and 14 (*n* = 3, *p < 0.05, ***p < 0.001). **(G, H)** Transmission electron microscopy showing podocyte-like cells (p) and proximal tubular epithelium-like cells (te) within the organoids at day 14. Note the foot processes (fp) anchored to the basement membrane-like profile (bm) and the oriented brush-like border (bb) (G: Scale = 2 μm; **(H)** Scale = 500 nm). **(I)** Schematic representation of kidney organoids (day 14).

We followed the three steps in the published protocol (Przepiorski et al., 2018) to promote differentiation of ESCs into a kidney organoid (Figure 1A) as detailed in the Methods section. We cultured the EBs derived from H9 iPSCs in “phase II” medium, and allowed the number of colonies to increase gradually from day 3 to day 8. On day 14, we observed the first discernible tubular profiles within the spheres (Figure 1B). The size of the kidney organoid was approximately 300 μm in diameter. The homogeneous pluripotency-associated markers (OCT4 and TRA-1-81) were expressed in the H9 colonies at passage 30 (Figure 1C), before organoid induction. However, the visible morphological diversity, such as discernible glomeruli and tubules, of the tissues (by H&E staining) appeared after 14 days of induction (Figure 1D). The phenotypic characterization of organoid cells suggested the formation of nephrons, the structural and functional units of the kidney, with distal end tubular (CDH1^+^), renal mesenchymal (MEIS1/2/3^+^), proximal tubule (LTL^+^), glomerulus foot (NPHS1^+^), proximal absorptive epithelial (LRP2^+^), collecting duct (GATA3), thick ascending limb (UMOD), and endothelial (CD31^+^) cells (Figure 1E). In addition, we observed a gradual increment in the expression of renal genes, including *SLC12A1*, *NPSH1*, *GATA3*, *LRP2,* and *CD31* by qRT-PCR during the first 2 weeks after the differentiation induction (Figure 1F), among these genes, the expression of *LRP2* reached its peak on the day8 and remained stable on the day14, indicative of a temporal maturation pattern of the kidney organoids. Moreover, we observed the ultrastructure of the organoids under transmission electron microscopy (TEM) and found distinctive podocyte-like cells with multiple foot processes anchoring to the basement membrane-like profiles and proximal epithelium-like cells with oriented brush-like protrusions (Figures 1G, H). These results confirm the successful establishment of kidney organoids resembling functional kidney tissue cells. These data collectively displayed that the organoid derived from hESC exhibited structural similarity to the actual kidney, including glomeruli, renal tubules, interstitial cells, and collecting ducts. (Figure 1I).

### 3.2 Development of SLC2A9 rs16890979 mutation, overexpression, and knockdown H9 cell lines

**FIGURE 2:**
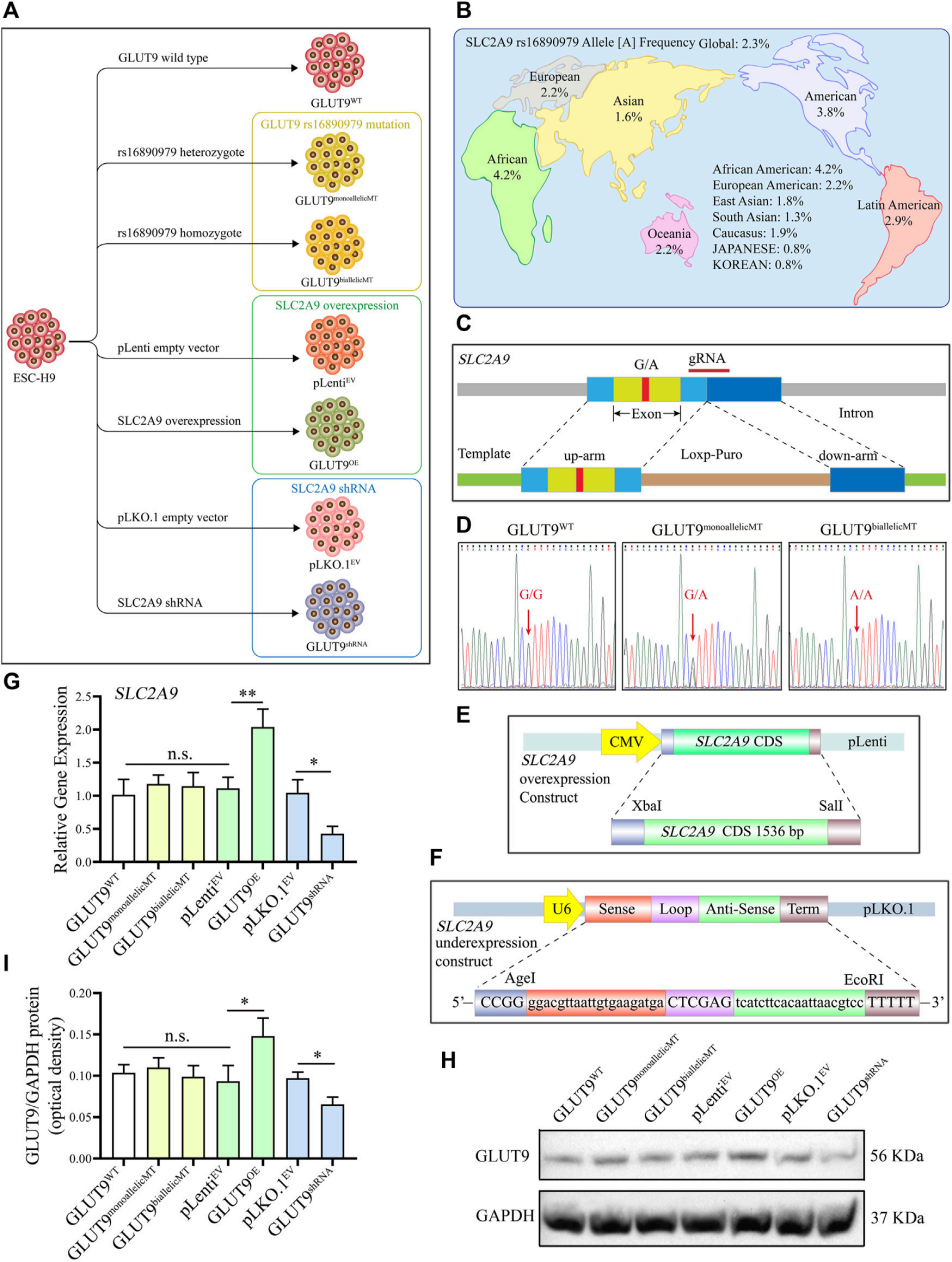
Development of GLUT9^rs16890979^, GLUT9^OE^, and GLUT9^shRNA^ H9 cell lines. **(A)** Schematic diagram showing the wildtype and genetically manipulated H9 cell lines used in this study. **(B)** Global frequencies and distribution of the SLC2A9 rs16890979 polymorphism according to the National Library of Medicine database. **(C)** Schematic diagram for the CRISPR/Cas9 homologous gene repair template we used. **(D)** Sequencing of wild-type and gene-edited *SLC2A9*. **(E)** Schematic diagram of the lentivirus plasmid to overexpress *SLC2A9*. **(F)** Schematic diagram of the shRNA lentivirus plasmid for targeting *SLC2A9*. **(G)** *SLC2A9* mRNA expression in each H9 cell line assessed by qRT-PCR. **(H, I)** SLC2A9 protein expression in each H9 cell line assessed by Western blotting.

To study the function of SLC2A9 rs16890079, we developed human H9 ESCs lines with SLC2A9 rs16890979 mutations, overexpression, or knockdown (Figure 2A). According to the data on the National Library of Medicine database, the incidence of the rs16890979 SNP in SLC2A9 is approximately 2.3% worldwide (Figure 2B). We used the CRISPR/Cas9 gene homology repair technique (Rong et al., 2014) to insert a point mutation in the wild type ESC-H9 cell line (H9^WT^) by site-editing *SLC2A9* to generate the SNP rs16890979, a heterozygous H9-GLUT9^monoallelicMT^ line, and a homozygous H9-GLUT9^biallelicMT^ line. Figure 2C shows the positions of the guide RNA (gRNA) and repair template in the gene homologous repair plasmid map. We verified the successful site-directed mutation changing G to A on exon 8 of the *SLC2A9* gene using Sanger sequencing (Figure 2D). As shown in the technique roadmap (Figures 2E, F), we developed stable H9 cell lines overexpressing (H9-GLUT9^OE^) or knocking down (GLUT9^shRNA^) the *SLC2A9* gene, together with their corresponding empty vector controls (H9-pLenit^EV^ and H9-pLKO.1^EV^) (Figure 2A) to elucidate the function of the *SLC2A9* gene. In addition, we performed qRT-PCR and Western blots to identify the *SLC2A9* gene expression profile in each cell line. Our results show that the *SLC2A9* gene mRNA and protein were significantly upregulated in GLUT9^OE^ and downregulated in GLUT9^shRNA^ relative to their counterparts (i.e., GLUT^WT^, GLUT9^monoallelicMT^, GLUT9^biallelicMT^, pLenit^EV^, and pLKO.1^EV^) (Figures 2G–I). All of the above-mentioned H9 cell lines presented normal ESC phenotypes (data not shown).

### 3.3 Phenotypic characteristics of kidney organoids derived from GLUT9^rs16890979^, GLUT 9^OE^, and GLUT 9^shRNA^ H9 ESCs under normal culture conditions

**FIGURE 3:**
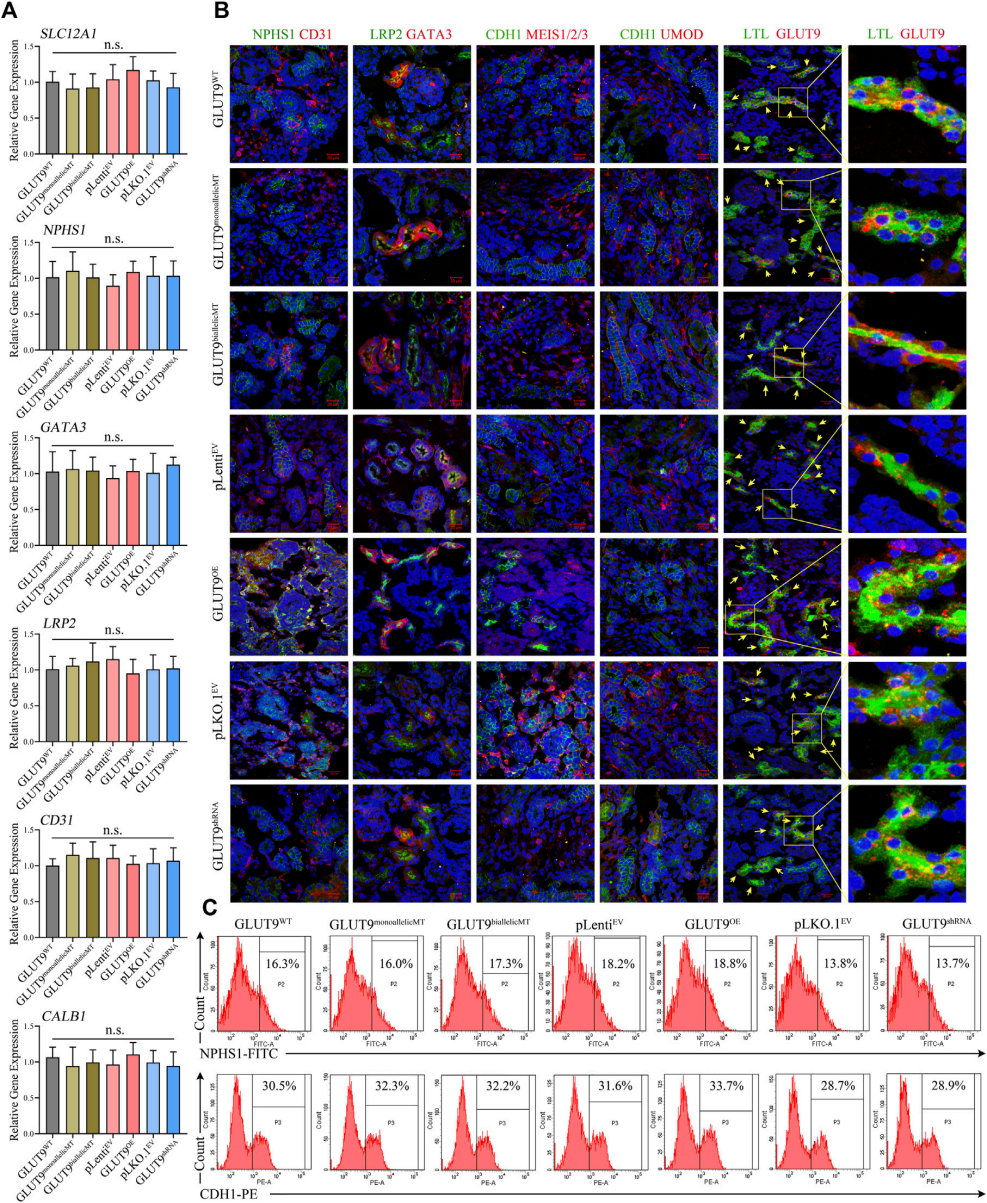
Phenotypic characterization of kidney organoids derived from H9^WT^, H9-GLUT9^monoallelicMT^, H9-GLUT9^biallelicMT^, H9-GLUT9^OE^, H9-GLUT9^shRNA^, H9-pLenit^EV^, and H9-pLKO.1^EV^. **(A)** Similar *SLC2A9* mRNA expressions among kidney organoids derived from each H9 cell line (*n* = 3, n. s not significant). **(B)** Immunofluorescence staining showing kidney tissue markers, including NPHS1, CD31, LPR2, GATA3, CDH1, MES1/2/3, UMOD, LTL, and SLC2A9 expressed in kidney organoids derived from each H9 cell line. Note the co-localization of GLUT9 and LTL in the tubular profiles. Scale bars, 20 μm; ×40 oil objective; DAPI, green fluorescence, and red fluorescence were excited by laser pulses of 350 nm, 488 nm and 555 nm, respectively. **(C)** FACS showing approximate proportions of NPHS1 or CDH1 immuno-positive cells within kidney organoids derived from each H9 cell line.

We induced all H9 cell lines, including H9-GLUT^WT^, H9-GLUT9^monoallelicMT^, H9-GLUT9^biallelicMT^, H9-GLUT9^OE^, H9-GLUT9^shRNA^, H9-pLenit^EV^, and H9-pLKO.1^EV^ to form kidney organoids using the method mentioned. After 14 days of differentiation, we detected the mRNA expressions of kidney tissue-specific genes, including *SLC12A1*, *NPHS1*, *GATA3*, *LRP2*, and *CD31* by qRT-PCR. We found similar mRNA expression levels among the organoids derived from different cell lines (Figure 3A). In addition, immunostaining showed that all H9-derived organoids expressed representative renal markers including NPHS1, CD31, LRP2, GATA3, UMOD, and LTL (Figure 3B). The expression of markers of tubular or glomerular morphology further validated the tissue structure within the organoids. Notably, we observed the co-localization of GLUT9 and LTL suggesting that GLUT9 was mainly expressed in the basolateral membranes of proximal tubules, as published (Augustin et al., 2004). Additionally, we were able to discern increasing and decreasing GLUT9 fluorescence intensities in the organoids of H9-SLC2A9^OE^ and H9-SLC2A9^shRNA^, respectively (Figure 3B), suggesting the correct functioning of our overexpression or RNA interference systems following organoid induction. We performed FACS to assess the ratio of selected types of cells within the organoids derived from different H9 cell lines. The proportion of the representative NPHS1 marker for immunolabeling of foot cells ranged between 13.8% and 18.8%, and that of the CDH1 marker for distal end tubule cells ranged between 28.7% and 33.7% in organoids with different H9 origins (Figure 3C). Thus, our results suggest that there were no significant phenotypic differences in the organoids derived from different H9 cell lines under normal culture conditions.

### 3.4 rs16890979 SNP reduces the absorption of UA in kidney organoids

**FIGURE 4:**
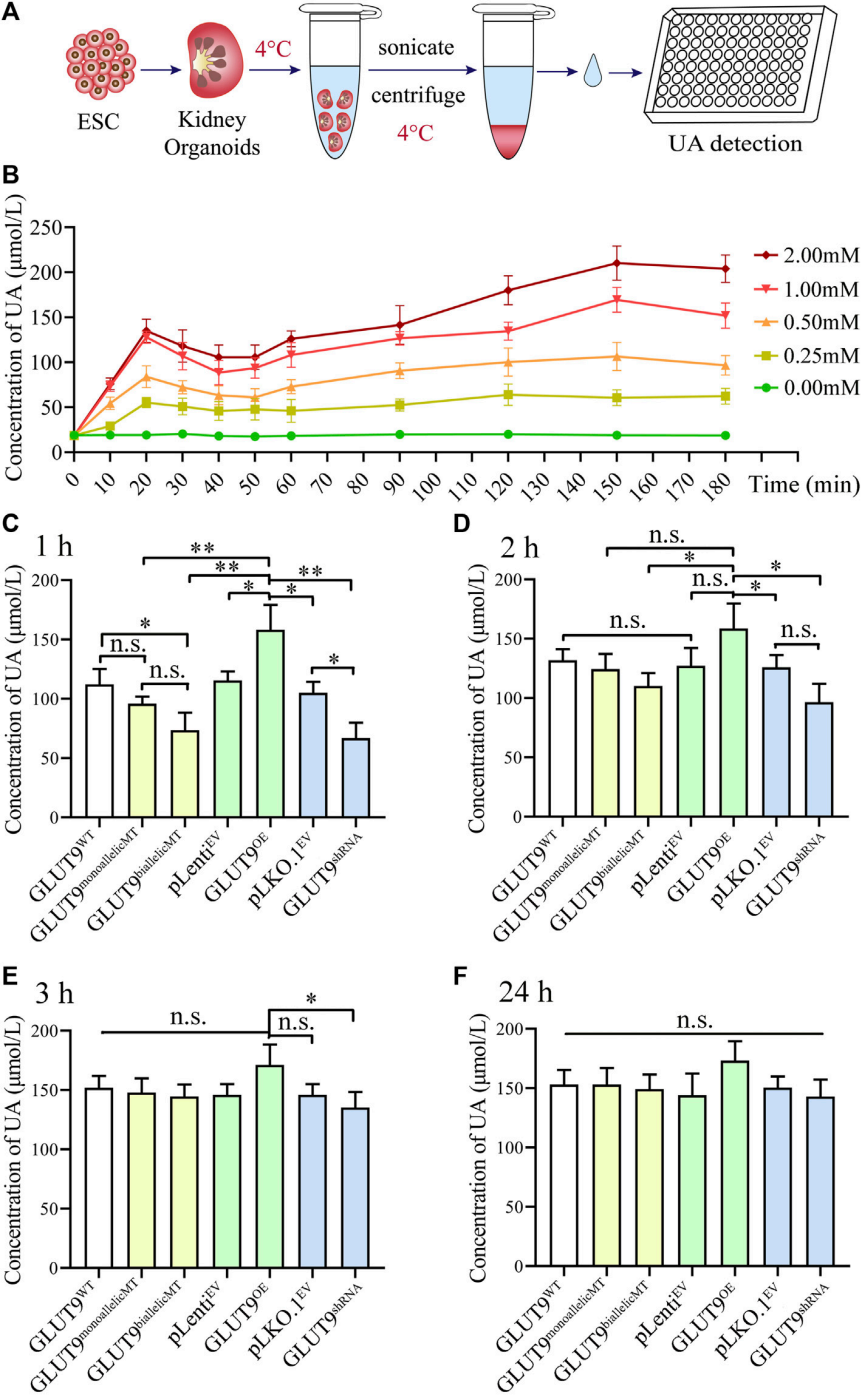
The rs16890979 SNP reduces the absorption of UA by kidney organoids. **(A)** A schematic diagram showing the procedures for the detection uric acid in kidney organoid. **(B)** Time lapse detection of UA concentration in GLUT9^WT^ kidney organoid after exposure to different dose of urate in culture medium. **(C–F)** UA concentrations retained in kidney organoids cultured in medium containing 1 mM urate for 1, 2, 3, and 24 h (*n* = 3, *p < 0.05, **p < 0.005, n. s not significant).

To investigate UA uptake by kidney organoids, we firstly established the baseline of UA absorption efficiency using GLUT9^WT^ kidney organoid. The results showed that when the kidney organoids were placed in UA medium (Figure 4A), the UA level within the kidney organoids rapidly peaked at around 20min after exposure, followed by a putative excretion of UA lasting for at least 30min. The UA level in the kidney organoids raised again at 50min and reached a plateau at around 2 h after exposure. At this time point the UA in the kidney organoids might have almost reached electro-chemical equilibrium (Figure 4B). Then we added 1 mM of urate into the culture system to mimic the UA level in the primary urine. We harvested, rinsed, and homogenized the organoids after incubation in UA medium for 1, 2, 3, and 24 h and calculated the UA amounts retained within the organoids. At these four time points, the UA concentration in the kidney organoids increased gradually, and the rate of UA uptake varied between clones (Figures 4C–F). Our results show that after incubation in urate medium for 1 h, the UA content within the organoids derived from H9-GLUT9^monoallelicMT^, H9-GLUT9^biallelicMT^, and H9-GLUT9^shRNA^ cell lines was lower than the contents in the rest of the cell lines (Figure 4C), suggesting that the rs16890979 SNP affects the cellular absorption of UA in a fashion similar to *SLC2A9* knockdown. By contrast, the UA retained increased significantly in the organoids with upregulated *SLC2A9* expression (H9-GLUT9^OE^) (Figure 4C). After incubating the organoids for 2 h in the UA medium (Figure 4D), the UA content within the organoids from each cell line was higher than that in their counterparts following 1 h of UA treatment (Supplementary Table S1). Notably, the UA content within organoids derived from H9-GLUT9^monoallelicMT^ and H9-GLUT9^biallelicMT^ increased to levels similar to those within the organoids derived from H9-GLUT9^WT^ or H9-GLUT9^OE^, indicating that UA absorption may reach a plateau after incubation in UA for 2 h. However, the retained UA within the organoids derived from H9-GLUT9^shRNA^ was still significantly lower than that in the organoids from H9-GLUT9^OE^. After 3 h, the UA concentration in kidney organoids in each experimental group did not increase further. We estimate that the maximum capacity for UA retention within the kidney organoids developed in this study was approximately 150 μmol/L because we found no further UA elevations during the period spanning hours 3 to 24 (Figures 4E, F).

We attempted to use molecular docking to predict the impact of rs16890979 on the protein structure of GLUT9 and the binding ability of GLUT9 to UA. The Laplace plot of protein structure and the overlap plot of PDB format are shown in Supplementary Figure S1A (GLUT9a) and Supplementary Figure S1B (GLUT9b). The results showed that the rs16890979 SNP had little effect on the structure of GLUT9 protein. The molecular docking results showed a slight decrease in the affinity between the GLUT9^rs16890979^ and UA compared to the GLUT9^WT^ (Supplementary Figure S2). The minimum binding energies between UA and GLUT9 was −5.65 kcal/mol to GLUT9a^WT^, −5.63 kcal/mol to GLUT9a^rs16890979^ (Supplementary Figure S2A, B), −5.69 kcal/mol to GLUT9b^WT^ and −5.31 kcal/mol to GLUT9b^rs16890979^ (Supplementary Figure S2C, D). The impact of rs16890979 SNP on the structure of GLUT9 protein and UA binding ability was minimal, indicating that there might be multiple mechanisms by which SNP affects the absorption of UA by kidney organoid.

### 3.5 rs16890979 does not confer additional resilience to environmental UA-induced EMT in kidney organoids

**FIGURE 5:**
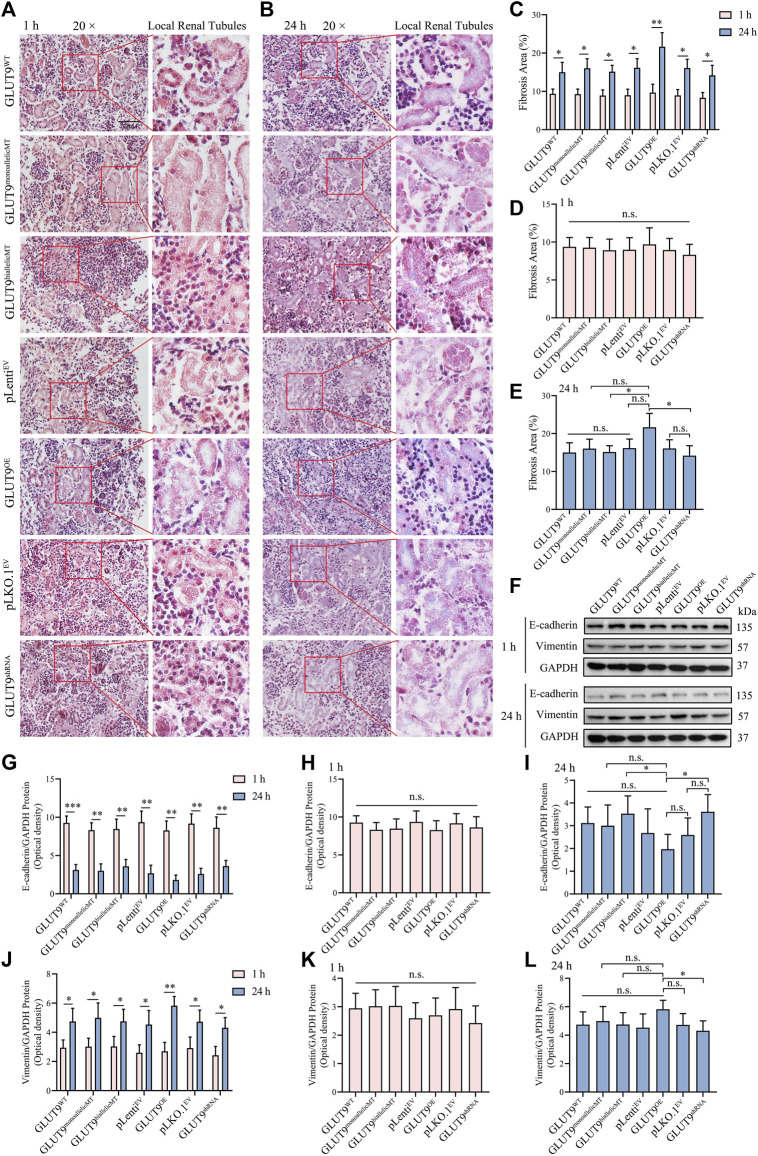
Hyperuricosuria induces EMT in kidney organoids. **(A, B)** Masson staining showing the lager blue areas of 24-h kidney organoids compared with those in 1-h kidney organoids (Scale bars, 50 μm; ×20 objective). **(C)** The quantitative Masson staining data show significant differences (*n* = 3, *p < 0.05, **p < 0.01). **(D)** The fibrosis extents in the kidney organoids of different experimental groups at 1 h were similar (n.s., not significantly). **(E)** At 24 h, the fibrosis extent in GLUT^OE^ kidney organoids was significantly higher than those in GLUT9^biallelicMT^ and GLUT9^shRNA^ organoids (*n* = 3, *p < 0.05; n. s., not significantly). **(F)** Western blot data showing that the expression of vimentin was higher and that of E-cadherin lower after 24 h than those after 1 h **(G–L)** Western blot (WB) quantitative data showing statistically significant differences (*n* = 3, *p < 0.05, **p < 0.01, ***p < 0.001, n. s., not significantly).

To study the effect of UA exposure on the kidney organoid phenotypes, we carried out Masson stainings to visualize collagen deposition within the treated organoids. Our results showed convergent upregulation of blue-stained areas in all organoids after 24 h of exposure to 1 mM urate in comparison with the relatively negligible blue-stained areas in organoids after only 1 h of exposure (Figures 5A–E; Supplementary Table S2), indicating collagen fibril production induction, a sign of epithelial-mesenchymal transition (EMT). The masson staining showed that at 24 h, the blue area is mainly presented at the renal tubular brush border (Figure 5B). It suggested that collagen fibrils may first occur at the brush like border of renal tubular cells. Gene editing resulted in the alteration of amino acid for both GLUT9a and GLUT9b. Theoretically both influx and efflux of UA by the tubular epithelium may be affected. However, due to the lack of blood supply to cargo the efflux UA in kidney organoid, whether the efflux may function properly is questionable. The Masson data showed EMT occurred in renal tubes, which might suggest the possibility of impairment secondary to accumulation of UA within the tubular epithelium (Figures 5A, B). However, at current stage it would be difficult to ascertain tissue distribution of absorbed UA within the organoid. We extracted the total protein of kidney organoids and analyzed the expression of the EMT-specific proteins vimentin and E-cadherin by Western blotting to confirm that the exposure to UA in the medium causes EMT in kidney organoids, and our results showed that after culture in UA medium for 24 h, the concentrations of UA in the kidney organoids of each experimental group increased, while the expressions of vimentin increased gradually and of E-cadherin decreased gradually, signaling EMTs (Figure 5F). Those statistically significant differences in the protein levels (Figures 5G–L; Supplementary Tables S3, S4) support our finding that continuous high UA stimulation leads to EMT in kidney organoids, a result consistent with the pathological characteristics seen in clinical hyperuricemic nephropathy. However, although rs16890979 reduced UA uptake in the kidney organoids, EMT still occurred within the parenchyma, and we found similar collagenous deposition and EMT-related protein expression levels in wildtype and SNP-containing organoids (Figures 5E,I and L; Supplementary Tables S3, S4).

## 4 Discussion

UA absorption in the proximal tube is a main factor for UA metabolism homeostasis in humans. *SLC2A9* is located on chromosome 4 P15 3-p16, it contains 13 exons and encodes both long and short types of GLUT9 (Phay et al., 2000; Augustin et al., 2004; Luscher et al., 2019) in charge of the transportation of UA from the primary urine into the renal tubular epithelial cells and eventually into the blood (Hattersley et al., 2008; Torres et al., 2014). GLUT9 is expressed at the topical and basolateral membrane of the proximal tubes (Luscher et al., 2019). GLUT9b is responsible for absorbing UA from the renal tubular lumen into renal tubular cells, while GLUT9a is responsible for transporting UA from the renal tubular cells to the renal interstitium or blood system. Thus completing the reabsorption process of UA. *SLC2A9* causes various independent pathogenic genetic effects, and its gene polymorphisms affect serum UA levels (Tu et al., 2010; Rule et al., 2011). Studies have reported that the rs16890979 SNP is associated with serum UA levels (Parsa et al., 2012; Torres and Puig, 2018). However, this same SNP has been reported to cause hyperuricemia (Cho et al., 2020); and hypouricemia (Dehghan et al., 2008; Hurba et al., 2014; Lee et al., 2018) depending on the geographical location and ethnicity of the population studied. Therefore, whether rs16890979 is a risk factor or an attenuator for hyperuricemia has been unclear.

We demonstrated that rs16890979 reduces UA absorption in human kidney organoids with defined genetic backgrounds and appropriate phenotypic characteristics. Although the exact molecular mechanisms remain obscure, we believe we were able to develop the first primary renal hypouricemia organoid model to address fundamental questions about the function of rs16890979 in human kidneys. Therefore, our study serves as a proof of concept for developing organoid-based solutions to determine the function of different SNPs on UA metabolism.

Indeed, the rapid development of kidney organoid technologies has led to reliable *in vitro* 3-dimensional models to simulate the physiological and pathological features of the human kidney (Nishinakamura, 2019) with resolutions at transcriptome (Low et al., 2019) and metabolome (Wang et al., 2021) levels. However, to the best of our knowledge no other study had applied an organoid model with defined genetic background to the study of UA metabolism before. We discovered the function of rs16890979 on UA metabolism in kidney organoids generated after a site-directed mutation of human H9 ESC line cells using CRISPR/Cas9 gene homology repair technology. We found kidney organoids with SNPs that downregulate UA absorption, in particular a homozygous G to A mutation, simulating the pathophysiology observed in patients with homozygous mutations, who present more severe hypouricemia than patients with heterozygous mutations (Parsa et al., 2012; Kanbay et al., 2022). This observation also agrees with the results of GWAS studies suggesting that this SNP causes hypouricemia in European populations (Dehghan et al., 2008; Lee et al., 2018). However, this finding contradicts the results of a Korean GWAS study showing that the same SNP may cause hyperuricemia (Cho et al., 2020). To address the function of GLUT9 in the kidney, we established organoids overexpressing or downregulating SLC2A9 to conduct experiments, and our results support the idea that GLUT9 is responsible for UA absorption. Therefore, regardless of the genetic background of kidney organoids, we believe that rs16890979 may result in abnormal UA reabsorption causing renal hypouricemia in patients. GLUT9 is expressed in the liver (UA production) and intestines (UA excretion) (Ruiz et al., 2018), but our kidney organoid model offers only renal UA metabolism insights. Studies are needed to clarify the cause of the different observed functions of rs16890979 among individuals of different ethnicities. Fused organoids or multiple-organoid-on-chip technologies based on our model may help the study of the combined effects of rs16890979 in kidney with liver and/or intestines. Furthermore, it is worthy to note that we have discovered the discrepancy between the biological effect observed in kidney organoid and homology modeling regarding the binding of GLUT9 and UA. The mild changes in UA affinity alteration between the SNP and wildtype as assessed by a computer simulation of molecular docking may suggest the possible existence of ancillary proteins or protein networks to chaperone GLUT9 in UA transportation. For examples, LAPTM4b, a binding partner for the Leu transporter, recruits LAT1-4F2hc (*SLC7A5-SLAC3A2*), leading to the uptake of Leu into lysosomes (Milkereit et al., 2015). By binding with a subunit of multiple amino acid transporters 4F2hc, the endocrine protein Girdin participates in the regulation of leucine entry into lysosome (Guan et al., 2018). Another example is the discovery of mutation in cysteine transporter subunits (b^0,+^AT-rBAT, *SLC3A1-SLC7A9*) in regulating cysteine metabolism. Symptomatic (cystinuria) or asymptomatic phenotypes are decided by the particular combination of heterozygosity/homozygosity across subunits (Calonge et al., 1994; Rajan et al., 1999). The disease is inherited kidney stones or cystinuria (Fotiadis et al., 2013). Additionally, the formation of large membrane complexes in the intestinal/renal epithelial transporters B^0^AT1 and B^0^AT3 and IMINO (*SLC6A19*, *18*, and *20*, respectively) and their complex interactions with the ancillary proteins ACE2 and collectrin in the small intestine and kidney, respectively participate in the etiology of Hartnup disorder (Kowalczuk et al., 2008; Camargo et al., 2009; Fairweather et al., 2012; Fairweather et al., 2015). These data taken together suggest the complexity in UA transportation, and the related ancillary proteins for GLUT9 are yet to be discovered. Nonetheless, kidney organoids established in this study may provide an alternative platform to reveal the functional interactions between GLUT9 with other possible ancillary proteins or protein networks.

Abnormal GLUT9 UA reabsorption leads to clinical complications including hyperuricourine-related renal calculi formation and exercise-induced acute renal failure (Nakayama et al., 2019). Thus, studying cytobiological changes in kidney organoids immersed in high UA media is important. Hyperuricosuria is usually difined as a UA GFR concentration higher than 0.56 mg/dL (approximately 0.03 mM GFR) (Saneian et al., 2022), the exact concentrations within the renal tubes and parenchyma remain unknown. It is considered that a serum UA concentration exceed of 6 mg/dL (360μM, 0.36 mM) is defined as HUA, while a serum UA concentration of 1 mM is relatively rare in the human body (Pascart and Lioté, 2018). The concentrations of UA applied *in vitro* to mimic hyperuricosuria conditions have usually been set at approximately the 1 mM (Liu et al., 2022; Shi et al., 2023) to 3 mM (Ding et al., 2022) levels. In this study, we found pronounced tissue impairment, presenting as increased renal fibrosis and EMT-related molecule upregulation, in organoids exposed to 1 mM UA for 24 h, demonstrating the vulnerability of the organoids to the UA challenge. However, despite the decreased UA absorption of organoids from H9-GLUT9^monoallelicMT^ and H9-GLUT9^biallelicMT^, their proximal tubular cells still presented UA-related impairment after exposure for 24 h. This may indicate the cellular target to study the mechanism of hyperuricosuria-related renal pathology. However, future studies may still need to use our organoid model to explore the exact molecular alteration in putative tissue cells to elucidate the mechanisms at play during hyperuricosuria-related renal pathologies, such as exercise-induced acute kidney injury (Miyamoto et al., 2022). Although we have confirmed that GLUT9 was mainly expressed in renal tubules of kidney organoids by immunofluorescence, it remains to be validated that the localization of GLUT9a and GLUT9b in organoid may be the same as they are in physiologic kidneys since the tissue disruption is relatively disorganized in the organoids. It is therefore desirable to design targeted probes to verify the tissue and cellular location of GLUT9a and GLUT9b (Kalsi et al., 2008; Fairweather et al., 2012). Additionally, the application of autoradiography such as [^14^C]-UA would be helpful to validate the exact cellular absorption and tissue distribution of UA by GLUT9 in the kidney organoids. Indeed, combination of advanced methodology may help to improve technical limitations of current kidney organoids and offer more insights to dissect kidney functions.

## 5 Conclusion

In conclusion, we developed a gene-editing kidney organoid protocol to study UA metabolism, and we showed that both heterozygous and homozygous rs16890979 SNP mutations led to reduced UA absorption. Our results from molecular, cellular, and organ levels support the correlation between this SNP and the renal hypouricemia observed in the clinic. Thus, our organoid model, based on the principle of synthetic biology (Trentesaux et al., 2023), may provide a valuable platform to further explore the function of SNPs and to assess the therapeutic effects of interventions against primary hypouricemia.

## Data Availability

The original contributions presented in the study are included in the article/Supplementary Material, further inquiries can be directed to the corresponding authors.

## Abbreviations

ANOVA, One-way analysis of variance; DMEM, Dulbecco’s modified Eagle’s medium; EMT, Epithelial-mesenchymal transition; ESCs, Embryonic stem cells; GWAS, Genome-wide association study; gRNA, Guide RNAs; HUA, Hyperuricemia; SNPs, Single nucleotide polymorphisms; OE, Overexpression; shRNA, short hairpin RNA; EBs, Embryoid bodies; RT, Room temperature; TEM, Transmission electron microscopy; UA, Uric acid

## References

[B1] Adachi S. I. Yoshizawa F. Yagasaki K. (2017). Assay systems for screening food and natural substances that have anti-hyperuricemic activity: uric acid production in cultured hepatocytes and purine bodies-induced hyperuricemic model mice. Cytotechnology 69 (3), 435–442. 10.1007/s10616-016-0005-z 27518104 PMC5461235

[B2] Anzai N. Ichida K. Jutabha P. Kimura T. Babu E. Jin C. J. (2008). Plasma urate level is directly regulated by a voltage-driven urate efflux transporter URATv1 (SLC2A9) in humans. J. Biol. Chem. 283 (40), 26834–26838. 10.1074/jbc.C800156200 18701466

[B3] Augustin R. Carayannopoulos M. O. Dowd L. O. Phay J. E. Moley J. F. Moley K. H. (2004). Identification and characterization of human glucose transporter-like protein-9 (GLUT9): alternative splicing alters trafficking. J. Biol. Chem. 279 (16), 16229–16236. 10.1074/jbc.M312226200 14739288

[B4] Balakumar P. Alqahtani A. Khan N. A. Mahadevan N. Dhanaraj S. A. (2020). Mechanistic insights into hyperuricemia-associated renal abnormalities with special emphasis on epithelial-to-mesenchymal transition: pathologic implications and putative pharmacologic targets. Pharmacol. Res. 161. 10.1016/j.phrs.2020.105209 32979505

[B5] Calonge M. J. Gasparini P. Chillarón J. Chillón M. Gallucci M. Rousaud F. (1994). Cystinuria caused by mutations in rBAT, a gene involved in the transport of cystine. Nat. Genet. 6 (4), 420–425. 10.1038/ng0494-420 8054986

[B6] Camargo S. M. R. Singer D. Makrides V. Huggel K. Pos K. M. Wagner C. A. (2009). Tissue-specific amino acid transporter partners ACE2 and collectrin differentially interact with Hartnup mutations. Gastroenterology 136 (3), 872–882. e873. 10.1053/j.gastro.2008.10.055 19185582 PMC7094282

[B7] Chiba T. Matsuo H. Nagamori S. Nakayama A. Kawamura Y. Shimizu S. (2014). Identification of a hypouricemia patient with SLC2A9 R380W, A pathogenic mutation for renal hypouricemia type 2. Nucleosides, Nucleotides Nucleic Acids 33 (4-6), 261–265. 10.1080/15257770.2013.857781 24940677

[B8] Cho S. K. Kim B. Myung W. Chang Y. Ryu S. Kim H. N. (2020). Polygenic analysis of the effect of common and low-frequency genetic variants on serum uric acid levels in Korean individuals. Sci. Rep. 10 (1), 9179. 10.1038/s41598-020-66064-z 32514006 PMC7280503

[B9] Dalbeth N. Gosling A. L. Gaffo A. Abhishek A. (2021). Gout. *Lancet* 397 (10287), 1843–1855. 10.1016/s0140-6736(21)00569-9 33798500

[B10] Dehghan A. Kottgen A. Yang Q. Hwang S. J. Kao W. L. Rivadeneira F. (2008). Association of three genetic loci with uric acid concentration and risk of gout: a genome-wide association study. Lancet 372 (9654), 1953–1961. 10.1016/S0140-6736(08)61343-4 18834626 PMC2803340

[B11] Ding Z. Zhao J. Wang X. Li W. Chen C. Yong C. (2022). Total extract of Abelmoschus manihot L. alleviates uric acid-induced renal tubular epithelial injury via inhibition of caspase-8/caspase-3/NLRP3/GSDME signaling. Front. Pharmacol. 13, 907980. 10.3389/fphar.2022.907980 36052125 PMC9424722

[B12] Dinour D. Gray N. K. Campbell S. Shu X. Sawyer L. Richardson W. (2010). Homozygous SLC2A9 mutations cause severe renal hypouricemia. J. Am. Soc. Nephrol. 21 (1), 64–72. 10.1681/asn.2009040406 19926891 PMC2799278

[B13] Dinour D. Gray N. K. Ganon L. Knox A. J. S. Shalev H. Sela B. A. (2011). Two novel homozygous SLC2A9 mutations cause renal hypouricemia type 2. Nephrol. Dial. Transplant. 27 (3), 1035–1041. 10.1093/ndt/gfr419 21810765

[B14] Doblado M. Moley K. H. (2009). Facilitative glucose transporter 9, a unique hexose and urate transporter. Am. J. Physiology-Endocrinology Metabolism 297 (4), E831–E835. 10.1152/ajpendo.00296.2009 PMC2763791 19797240

[B15] Döring A. Gieger C. Mehta D. Gohlke H. Prokisch H. Coassin S. (2008). SLC2A9 influences uric acid concentrations with pronounced sex-specific effects. Nat. Genet. 40 (4), 430–436. 10.1038/ng.107 18327256

[B16] Fairweather S. J. Bröer A. O'Mara M. L. Bröer S. (2012). Intestinal peptidases form functional complexes with the neutral amino acid transporter B0AT1. Biochem. J. 446 (1), 135–148. 10.1042/bj20120307 22677001 PMC3408045

[B17] Fairweather S. J. Bröer A. Subramanian N. Tumer E. Cheng QI Schmoll D. (2015). Molecular basis for the interaction of the mammalian amino acid transporters B0AT1 and B0AT3 with their ancillary protein collectrin. J. Biol. Chem. 290 (40), 24308–24325. 10.1074/jbc.M115.648519 26240152 PMC4591816

[B18] Fotiadis D. Kanai Y. Palacín M. (2013). The SLC3 and SLC7 families of amino acid transporters. Mol. Asp. Med. 34 (2-3), 139–158. 10.1016/j.mam.2012.10.007 23506863

[B19] Guan K.-L. Weng L. Han Y.-P. Enomoto A. Kitaura Y. Nagamori S. (2018). Negative regulation of amino acid signaling by MAPK-regulated 4F2hc/Girdin complex. PLoS Biol. 16 (3). 10.1371/journal.pbio.2005090 PMC5868845 29538402

[B20] Halperin Kuhns V. L. Woodward O. M. (2021). Urate transport in health and disease. Best. Pract. Res. Clin. Rheumatol. 35 (4), 101717. 10.1016/j.berh.2021.101717 34690083 PMC8678298

[B21] Hattersley A. Caulfield M. J. Munroe P. B. O'Neill D. Witkowska K. C. Fadi J. (2008). SLC2A9 is a high-capacity urate transporter in humans. PLoS Med. 5 (10). 10.1371/journal.pmed.0050197 PMC2561076 18842065

[B22] Hollis‐Moffatt J. E. Xu X. Dalbeth N. Merriman M. E. Topless R. Waddell C. (2009). Role of the urate transporter SLC2A9 gene in susceptibility to gout in New Zealand Māori, Pacific Island, and Caucasian case–control sample sets. Arthritis Rheum. 60 (11), 3485–3492. 10.1002/art.24938 19877038

[B23] Hurba O. Mancikova A. Krylov V. Pavlikova M. Pavelka K. Stiburkova B. (2014). Complex analysis of urate transporters SLC2A9, SLC22A12 and functional characterization of non-synonymous allelic variants of GLUT9 in the Czech population: no evidence of effect on hyperuricemia and gout. PLoS One 9 (9), e107902. 10.1371/journal.pone.0107902 25268603 PMC4182324

[B24] Kalsi K. K. Baker E. H. Medina R. A. Rice S. Wood D. M. Ratoff J. C. (2008). Apical and basolateral localisation of GLUT2 transporters in human lung epithelial cells. Pflügers Archiv - Eur. J. Physiology 456 (5), 991–1003. 10.1007/s00424-008-0459-8 18239936 PMC2480509

[B25] Kanbay M. Xhaard C. Le Floch E. Dandine-Roulland C. Girerd N. Ferreira J. P. (2022). Weak association between genetic markers of hyperuricemia and cardiorenal outcomes: insights from the STANISLAS study cohort with a 20-year follow-up. J. Am. Heart Assoc. 11 (9), e023301. 10.1161/JAHA.121.023301 35470676 PMC9238600

[B26] Keenan R. T. (2020). The biology of urate. Semin. Arthritis Rheum. 50 (3S), S2–S10. 10.1016/j.semarthrit.2020.04.007 32620198

[B27] Kottgen A. Albrecht E. Teumer A. Vitart V. Krumsiek J. Hundertmark C. (2013). Genome-wide association analyses identify 18 new loci associated with serum urate concentrations. Nat. Genet. 45 (2), 145–154. 10.1038/ng.2500 23263486 PMC3663712

[B28] Kowalczuk S. Bröer A. Tietze N. Vanslambrouck J. M. Rasko J. E. J. Bröer S. (2008). A protein complex in the brush‐border membrane explains a Hartnup disorder allele. FASEB J. 22 (8), 2880–2887. 10.1096/fj.08-107300 18424768

[B29] Lee H. A. Park B. H. Park E. A. Cho S. J. Kim H. S. Park H. (2018). Long-term effects of the SLC2A9 G844A and SLC22A12 C246T variants on serum uric acid concentrations in children. BMC Pediatr. 18 (1), 296. 10.1186/s12887-018-1272-y 30189835 PMC6127956

[B30] Li X. Yan Z. Tian J. Zhang X. Han H. Ye F. (2019). Urate transporter URAT1 in hyperuricemia: new insights from hyperuricemic models. Ann. Clin. Lab. Sci. 49 (6), 756–762. 31882426

[B31] Liu N. Wang L. Yang T. Xiong C. Xu L. Shi Y. (2015). EGF receptor inhibition alleviates hyperuricemic nephropathy. J. Am. Soc. Nephrol. 26 (11), 2716–2729. 10.1681/asn.2014080793 25788532 PMC4625671

[B32] Liu X. Wang M. Jiang T. He J. Fu X. Xu Y. (2019). Ido1 maintains pluripotency of primed human embryonic stem cells by promoting glycolysis. Stem Cells 37 (9), 1158–1165. 10.1002/stem.3044 31145821

[B33] Liu Y. Gong S. Li K. Wu G. Zheng X. Zheng J. (2022). Coptisine protects against hyperuricemic nephropathy through alleviating inflammation, oxidative stress and mitochondrial apoptosis via PI3K/Akt signaling pathway. Biomed. Pharmacother. 156, 113941. 10.1016/j.biopha.2022.113941 36411660

[B34] Low J. H. Li P. Chew E. G. Y. Zhou B. Suzuki K. Zhang T. (2019). Generation of human PSC-derived kidney organoids with patterned nephron segments and a *de novo* vascular network. Cell Stem Cell 25 (3), 373–387. e379. 10.1016/j.stem.2019.06.009 31303547 PMC6731150

[B35] Luscher B. P. Surbek D. V. Clemencon B. Huang X. Albrecht C. Marini C. (2019). Different pharmacological properties of GLUT9a and GLUT9b: potential implications in preeclampsia. Cell Physiol. Biochem. 53 (3), 508–517. 10.33594/000000154 31502429

[B36] Matsuo H. Chiba T. Nagamori S. Nakayama A. Domoto H. Phetdee K. (2008). Mutations in glucose transporter 9 gene SLC2A9 cause renal hypouricemia. Am. J. Hum. Genet. 83 (6), 744–751. 10.1016/j.ajhg.2008.11.001 19026395 PMC2668068

[B37] McArdle P. F. Parsa A. Chang Y. P. Weir M. R. O'Connell J. R. Mitchell B. D. (2008). Association of a common nonsynonymous variant in GLUT9 with serum uric acid levels in old order amish. Arthritis Rheum. 58 (9), 2874–2881. 10.1002/art.23752 18759275 PMC2779583

[B38] Meng Q. Yue J. Shang M. Shan Q. Qi J. Mao Z. (2015). Correlation of GLUT9 polymorphisms with gout risk. Medicine 94 (44). 10.1097/md.0000000000001742 PMC4915872 26554771

[B39] Milkereit R. Persaud A. Vanoaica L. Guetg A. Verrey F. Rotin D. (2015). LAPTM4b recruits the LAT1-4F2hc Leu transporter to lysosomes and promotes mTORC1 activation. Nat. Commun. 6 (1). 10.1038/ncomms8250 PMC4455107 25998567

[B40] Miyamoto D. Sato N. Nagata K. Sakai Y. Sugihara H. Ohashi Y. (2022). Analysis of purine metabolism to elucidate the pathogenesis of acute kidney injury in renal hypouricemia. Biomedicines 10 (7). 10.3390/biomedicines10071584 PMC9312704 35884889

[B41] Mobasheri A. Neama G. Bell S. Richardson S. Carter S. D. (2013). Human articular chondrocytes express three facilitative glucose transporter isoforms: glut1, Glut3 and Glut9. Cell Biol. Int. 26 (3), 297–300. 10.1006/cbir.2001.0850 11991658

[B42] Morizane R. Lam A. Q. Freedman B. S. Kishi S. Valerius M. T. Bonventre J. V. (2015). Nephron organoids derived from human pluripotent stem cells model kidney development and injury. Nat. Biotechnol. 33 (11), 1193–1200. 10.1038/nbt.3392 26458176 PMC4747858

[B43] Morris G. M. Huey R. Lindstrom W. Sanner M. F. Belew R. K. Goodsell D. S. (2009). AutoDock4 and AutoDockTools4: automated docking with selective receptor flexibility. J. Comput. Chem. 30 (16), 2785–2791. 10.1002/jcc.21256 19399780 PMC2760638

[B44] Nakayama A. Matsuo H. Ohtahara A. Ogino K. Hakoda M. Hamada T. (2019). Clinical practice guideline for renal hypouricemia (1st edition). Hum. Cell 32 (2), 83–87. 10.1007/s13577-019-00239-3 30783949 PMC6437292

[B45] Nishinakamura R. (2019). Human kidney organoids: progress and remaining challenges. Nat. Rev. Nephrol. 15 (10), 613–624. 10.1038/s41581-019-0176-x 31383997

[B46] Parsa A. Brown E. Weir M. R. Fink J. C. Shuldiner A. R. Mitchell B. D. (2012). Genotype-based changes in serum uric acid affect blood pressure. Kidney Int. 81 (5), 502–507. 10.1038/ki.2011.414 22189840 PMC3640827

[B47] Pascart T. Lioté F. (2018). Gout: state of the art after a decade of developments. Rheumatology. 10.1093/rheumatology/key002 29547895

[B48] Phay J. E. Hussain H. B. Moley J. F. (2000). Cloning and expression analysis of a novel member of the facilitative glucose transporter family, SLC2A9 (GLUT9). Genomics 66 (2), 217–220. 10.1006/geno.2000.6195 10860667

[B49] Przepiorski A. Sander V. Tran T. Hollywood J. A. Sorrenson B. Shih J. H. (2018). A simple bioreactor-based method to generate kidney organoids from pluripotent stem cells. Stem Cell Rep. 11 (2), 470–484. 10.1016/j.stemcr.2018.06.018 PMC6092837 30033089

[B50] Rajan D. P. Kekuda R. Huang W. Wang H. Devoe L. D. Leibach F. H. (1999). Cloning and expression of a b0,+-like amino acid transporter functioning as a heterodimer with 4F2hc instead of rBAT. J. Biol. Chem. 274 (41), 29005–29010. 10.1074/jbc.274.41.29005 10506149

[B51] Rong Z. Zhu S. Xu Y. Fu X. (2014). Homologous recombination in human embryonic stem cells using CRISPR/Cas9 nickase and a long DNA donor template. Protein Cell 5 (4), 258–260. 10.1007/s13238-014-0032-5 24622843 PMC3978163

[B52] Ruiz A. Gautschi I. Schild L. Bonny O. (2018). Human mutations in SLC2A9 (Glut9) affect transport capacity for urate. Front. Physiol. 9. 10.3389/fphys.2018.00476 PMC6016318 29967582

[B53] Rule A. D. de Andrade M. Matsumoto M. Mosley T. H. Kardia S. Turner S. T. (2011). Association between SLC2A9 transporter gene variants and uric acid phenotypes in African American and white families. Rheumatol. Oxf. 50 (5), 871–878. 10.1093/rheumatology/keq425 PMC3077913 21186168

[B54] Saneian H. Esteki B. Bozorgzad M. Famouri F. Mehrkash M. Khademian M. (2022). Hyperuricosuria and hypercalciuria, probable etiologies of functional abdominal pain: a case-control study. J. Res. Med. Sci. 27, 4. 10.4103/jrms.JRMS_424_20 35342445 PMC8943580

[B55] Shi X. Zhuang L. Zhai Z. He Y. Sun E. (2023). Polydatin protects against gouty nephropathy by inhibiting renal tubular cell pyroptosis. Int. J. Rheum. Dis. 26 (1), 116–123. 10.1111/1756-185X.14463 36328529

[B56] Takahashi T. Beppu T. Hidaka Y. Hosoya T. (2021). Uric acid-lowering effect of dotinurad, a novel selective urate reabsorption inhibitor, in hypertensive patients with gout or asymptomatic hyperuricemia: a pooled analysis of individual participant data in phase II and III trials. Clin. Exp. Hypertens. 43 (8), 730–741. 10.1080/10641963.2021.1950752 34425059

[B57] Tin A. Marten J. Kuhns H. Victoria L. Li Y. Wuttke M. (2019). Target genes, variants, tissues and transcriptional pathways influencing human serum urate levels. Nat. Genet. 51 (10), 1459–1474. 10.1038/s41588-019-0504-x 31578528 PMC6858555

[B58] Torres R. J. de Miguel E. Bailen R. Banegas J. R. Puig J. G. (2014). Tubular urate transporter gene polymorphisms differentiate patients with gout who have normal and decreased urinary uric acid excretion. J. Rheumatol. 41 (9), 1863–1870. 10.3899/jrheum.140126 25128519

[B59] Torres R. J. Puig J. G. (2018). GLUT9 influences uric acid concentration in patients with Lesch-Nyhan disease. Int. J. Rheum. Dis. 21 (6), 1270–1276. 10.1111/1756-185X.13323 29879316

[B60] Trentesaux C. Yamada T. Klein O. D. Lim W. A. (2023). Harnessing synthetic biology to engineer organoids and tissues. Cell Stem Cell 30 (1), 10–19. 10.1016/j.stem.2022.12.013 36608674 PMC11684341

[B61] Tu H. P. Chen C. J. Tovosia S. Ko A. M. Lee C. H. Ou T. T. (2010). Associations of a non-synonymous variant in SLC2A9 with gouty arthritis and uric acid levels in Han Chinese subjects and Solomon Islanders. Ann. Rheum. Dis. 69 (5), 887–890. 10.1136/ard.2009.113357 19723617

[B62] Vitart V. Rudan I. Hayward C. Gray N. K. Floyd J. Palmer C. N. A. (2008). SLC2A9 is a newly identified urate transporter influencing serum urate concentration, urate excretion and gout. Nat. Genet. 40 (4), 437–442. 10.1038/ng.106 18327257

[B63] Wang Q. Xiong Y. Zhang S. Sui Y. Yu C. Liu P. (2021). The dynamics of metabolic characterization in iPSC-derived kidney organoid differentiation via a comparative omics approach. Front. Genet. 12, 632810. 10.3389/fgene.2021.632810 33643392 PMC7902935

[B64] Waterhouse A. Bertoni M. Bienert S. Studer G. Tauriello G. Gumienny R. (2018). SWISS-MODEL: homology modelling of protein structures and complexes. Nucleic Acids Res. 46 (W1), W296–W303. 10.1093/nar/gky427 29788355 PMC6030848

[B65] Wu H. Uchimura K. Donnelly E. L. Kirita Y. Morris S. A. Humphreys B. D. (2018). Comparative analysis and refinement of human PSC-derived kidney organoid differentiation with single-cell transcriptomics. Cell Stem Cell 23 (6), 869–881. e868. 10.1016/j.stem.2018.10.010 30449713 PMC6324730

[B66] Wu S. Yan M. Liu J. Li Y. Tian R. Li C. (2023). Clerodendranthus spicatus inhibits epithelial–mesenchymal transition of renal tubular cells through the NF-κB/Snail signalling pathway in hyperuricaemia nephropathy. Pharm. Biol. 61 (1), 1274–1285. 10.1080/13880209.2023.2243086 37599625 PMC10443970

[B67] Yan B. Liu D. Zhu J. Pang X. (2019). The effects of hyperuricemia on the differentiation and proliferation of osteoblasts and vascular smooth muscle cells are implicated in the elevated risk of osteopenia and vascular calcification in gout: an *in vivo* and *in vitro* analysis. J. Cell. Biochem. 120 (12), 19660–19672. 10.1002/jcb.29272 31407397

