# Supplementary material for: SLC2A9 rs16890979 reduces uric acid absorption by kidney organoids
Source: Front Cell Dev Biol. 2024 Jan 10;11:1268226. doi: 10.3389/fcell.2023.1268226 (PMC10806012; doi:10.3389/fcell.2023.1268226)
Supplement: Supplementary file 1 [file DataSheet1.docx]

Supplementary Material

**Supplementary Table 1. Comparison of urate concentration (mean ± SD, %) among different groups.**

| Gruops | n | 1 h | 2 h | 3 h | 24 h |
| --- | --- | --- | --- | --- | --- |
| ^a^GLUT9^WT^ | 3 | 112.31 ± 12.92 | 132.01 ± 9.20 | 151.62 ± 9.70 | 153.01 ± 12.93 |
| ^b^GLUT9^monoallelicMT^ | 3 | 95.89 ± 6.02 | 124.53 ± 12.74 | 147.76 ± 11.96 | 153.01 ± 14.04 |
| ^c^GLUT9^biallelicMT^ | 3 | 73.56 ± 14.92 | 110.24 ± 10.80 | 144.59 ± 10.13 | 149.20 ± 12.39 |
| ^d^pLenti^EV^ | 3 | 115.64 ± 7.46 | 127.23 ± 15.04 | 145.84 ± 9.29 | 144.13 ± 18.30 |
| ^e^GLUT9^OE^ | 3 | 158.33 ± 20.88 | 158.52 ± 21.24 | 171.17 ± 17.14 | 173.33 ± 16.27 |
| ^f^pLKO.1^EV^ | 3 | 105.10 ± 9.31 | 125.93 ± 10.47 | 145.56 ± 9.03 | 150.47 ± 9.52 |
| ^g^GLUT9^shRNA^ | 3 | 66.99 ± 12.92 | 96.60 ± 15.45 | 135.19 ± 13.05 | 142.86 ± 14.38 |

One-way ANOVA test was used to show the statistical difference.

1 h: a *VS*. c, d *VS*. e, e *VS*. f, f VS. g *P*<0.05; b *VS*. e, c *VS*. e, e VS. g, *P*<0.01; a *VS*. b, b *VS*. c *P*>0.05.

2 h: c *VS*. e, e *VS.* f, e *VS*. g *P*<0.05; a *VS*. b, a *VS*. c, a *VS*. d, d *VS*. e, f *VS*. g *P*>0.05.

3 h: e *VS*. g, *P*<0.05; No significant difference in pairwise comparison between other groups.

24 h: No significant difference in pairwise comparison between groups.

**Supplementary Table 2. Comparison of fibrosis area (mean ± SD, %) in Masson data among different groups.**

| Gruops | n | 1 h | 24 h |
| --- | --- | --- | --- |
| ^a^GLUT9^WT^ | 3 | 9.36 ± 1.25 | 15.00 ± 2.58 |
| ^b^GLUT9^monoallelicMT^ | 3 | 9.26 ± 1.36 | 16.04 ± 2.49 |
| ^c^GLUT9^biallelicMT^ | 3 | 8.92 ± 1.47 | 15.15 ± 1.68 |
| ^d^pLenti^EV^ | 3 | 8.98 ± 1.60 | 16.15 ± 2.42 |
| ^e^GLUT9^OE^ | 3 | 9.70 ± 2.20 | 21.65 ± 3.68 |
| ^f^pLKO.1^EV^ | 3 | 8.94 ± 1.54 | 16.08 ± 2.30 |
| ^g^GLUT9^shRNA^ | 3 | 8.32 ± 1.39 | 14.20 ± 2.60 |

Two-way ANOVA test was used to show the statistical difference.

1 h: No significant difference in pairwise comparison between groups.

24 h: c *VS*. e (*t*=3.72), e *VS*. g (*t*=4.26), *P*<0.05; No significant difference in pairwise comparison between other groups.

**Supplementary Table 3. Comparison of E-cadherin/GAPDH (mean ± SD, %) in WB data among different groups.**

| Gruops | n | 1 h | 24 h |
| --- | --- | --- | --- |
| ^a^GLUT9^WT^ | 3 | 9.28 ± 0.90 | 3.12 ± 0.71 |
| ^b^GLUT9^monoallelicMT^ | 3 | 8.32 ± 0.96 | 3.00 ± 0.92 |
| ^c^GLUT9^biallelicMT^ | 3 | 8.47 ± 1.29 | 3.55 ± 0.77 |
| ^d^pLenti^EV^ | 3 | 9.36 ± 1.45 | 2.68 ± 1.06 |
| ^e^GLUT9^OE^ | 3 | 8.28 ± 1.23 | 1.94 ± 0.61 |
| ^f^pLKO.1^EV^ | 3 | 9.17 ± 1.27 | 2.59 ± 0.75 |
| ^g^GLUT9^shRNA^ | 3 | 8.64 ± 1.41 | 3.61 ± 0.76 |

Two-way ANOVA test was used to show the statistical difference.

1 h: No significant difference in pairwise comparison between groups.

24 h: c *VS*. e (*t*=1.84), e *VS*. g (*t*=1.93), *P*<0.05; No significant difference in pairwise comparison between other groups.

**Supplementary Table 4. Comparison of Vimentin/GAPDH (mean ± SD, %) in WB data among different groups.**

| Gruops | n | 1 h | 24 h |
| --- | --- | --- | --- |
| ^a^GLUT9^WT^ | 3 | 2.94 ± 0.53 | 4.74 ± 0.91 |
| ^b^GLUT9^monoallelicMT^ | 3 | 3.02 ± 0.58 | 4.99 ± 1.03 |
| ^c^GLUT9^biallelicMT^ | 3 | 3.02 ± 0.69 | 4.75 ± 0.83 |
| ^d^pLenti^EV^ | 3 | 2.59 ± 0.55 | 4.53 ± 0.97 |
| ^e^GLUT9^OE^ | 3 | 2.70 ± 0.61 | 5.82 ± 0.63 |
| ^f^pLKO.1^EV^ | 3 | 2.92 ± 0.76 | 4.72 ± 0.79 |
| ^g^GLUT9^shRNA^ | 3 | 2.43 ± 0.61 | 4.31 ± 0.70 |

Two-way ANOVA test was used to show the statistical difference.

1 h: No significant difference in pairwise comparison between groups.

24 h: e *VS*. g (*t*=2.49), *P*<0.05; No significant difference in pairwise comparison between other groups.


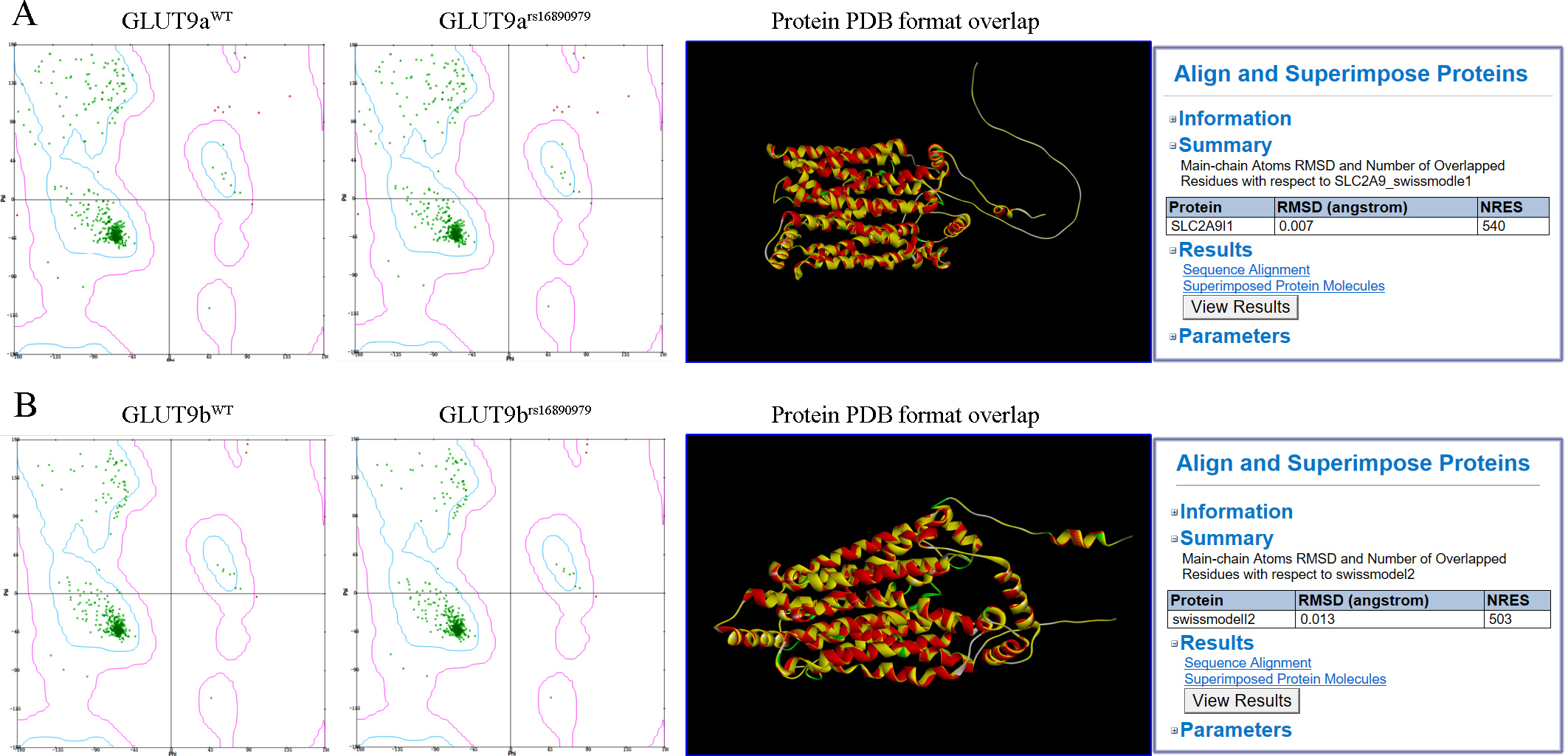


Supplementary Figure 1. homologous modeling of human SLC2A9 (GLUT9) protein. The laplace plot of protein structure and the overlap plot of PDB format, GLUT9a (**A**) and GLUT9b (**B**).


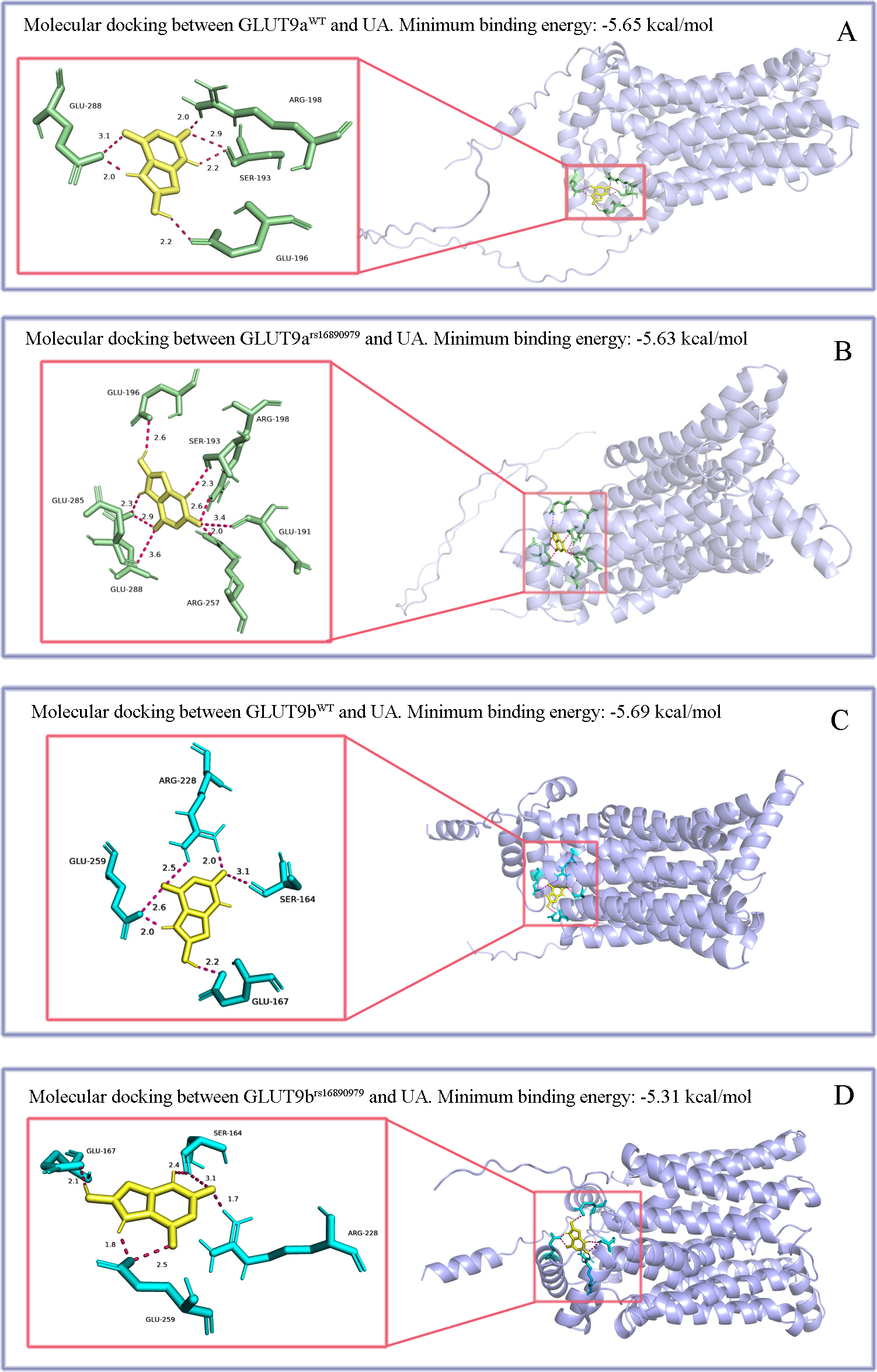


Supplementary Figure 2. UA and GLUT9 molecular docking prediction. The molecular docking results showed a slight decrease in the affinity between the GLUT9^rs16890979^ and UA compared to the GLUT9^WT^. The minimum binding energies between UA and GLUT9 was -5.65 kcal/mol to GLUT9a^WT^, -5.63 kcal/mol to GLUT9a^rs16890979^ (**A, B**), -5.69 kcal/mol to GLUT9b^WT^ and -5.31 kcal/mol to GLUT9b^rs16890979^ (**C, D**).
